# Interlayer Registry Index of Layered Transition Metal Dichalcogenides

**DOI:** 10.1021/acs.jpclett.1c04202

**Published:** 2022-04-08

**Authors:** Wei Cao, Oded Hod, Michael Urbakh

**Affiliations:** Department of Physical Chemistry, School of Chemistry, The Raymond and Beverly Sackler Faculty of Exact Sciences and The Sackler Center for Computational Molecular and Materials Science, Tel Aviv University, Tel Aviv 6997801, Israel

## Abstract

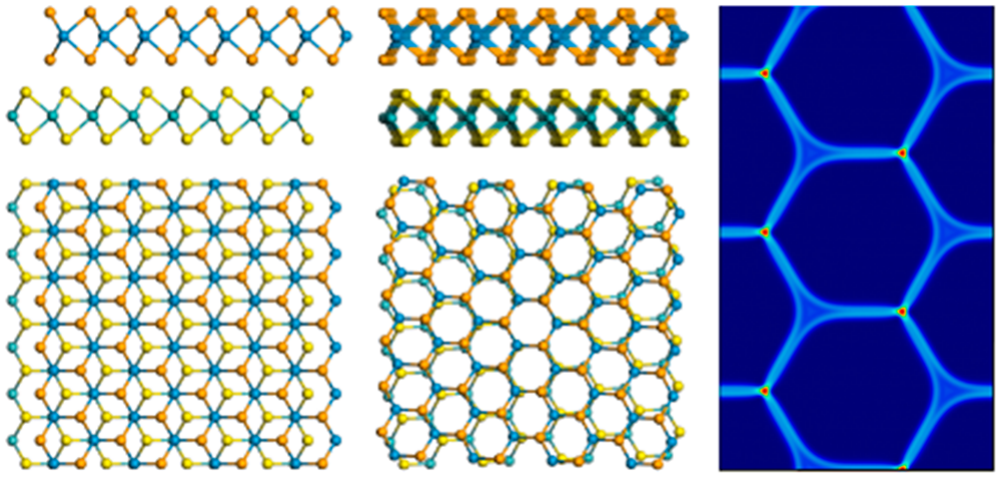

Inspired by the fascinating electronic properties of twisted transition metal dichalcogenides, we extend the registry index approach to quantify the interlayer commensurability of homogeneous and heterogeneous interfaces of MoS_2_, WS_2_, MoSe_2_, and WSe_2_. The developed geometric measure provides quantitative information about their sliding energy landscape with vast mechanical and tribological implications. Furthermore, the registry index is highly suitable for characterizing surface reconstruction in twisted transition metal dichalcogenide interfaces that dictates their intricate electronic and ferroelectric properties. The simple and intuitive nature of the registry index marks it as a powerful computational tool for studying the fascinating physical phenomena demonstrated by these materials.

The interlayer registry of two-dimensional van der Waals (vdW) layered materials has a dramatic effect on their structural and electronic properties. At small twist angles (θ ≈ 1°), for example, the lattice reconstruction at the vdW interface results in multiple commensurate stacking domains separated by boundary ridges,^1^ reflecting the intricate balance between interlayer vdW interactions and intralayer elasticity. In turn, such systems exhibit unique electronic transport properties, such as magic angle superconductivity^2^ and peculiar transversal current peaks.^3^ Beyond graphene, hexagonal boron nitride (*h*-BN) also exhibits extraordinary registry-dependent electronic phenomena. A rotation by 60° from the optimal AA′ stacking of *h*-BN could break the lattice centrosymmetry, giving rise to spontaneous charge polarization along the normal direction to the surface.^4−6^ An additional twist angle of ∼0.2° results in similar surface reconstruction, giving rise to stable moiré ferroelectric domains, whose polarization can be tuned via an external electric field.^4^

Another commensurability-related physical phenomenon appearing in twisted layered material interfaces is structural superlubricity, a state of ultralow friction and wear occurring at incommensurate rigid junctions.^7−10^ This intriguing phenomenon was initially demonstrated for homogeneous nanoscale graphitic interfaces, showing negligible friction at large twist angles.^11^ Such interfaces, however, are unstable and tend to dynamically rotate and lock into a commensurate high-friction state.^12−14^ This, in turn, can be remedied by considering heterogeneous junctions, such as those formed between graphene and *h*-BN, which were found to sustain robust superlubricity, immune to interfacial rotations under ambient conditions even at the microscale.^15−17^

Exciting registry-dependent physical phenomena were also observed in interfaces of layered transition metal dichalcogenides (TMDs) that, unlike graphite and *h*-BN, possess a 3-fold sublayer structure.^18,19^ These include the formation of flat electronic bands,^20^ the appearance of moiré exciton minibands,^21^ charge transfer and band alignment,^22^ and the emergence of deep moiré potentials.^23^ Similar to the case of graphene and *h*-BN junctions, imposing a small twist angle between the interfacing layers results in atomic reconstruction, where hexagonal and triangular domains are found for the antiparallel (AP) and parallel (P) stacking configurations, respectively.^24,25^ At large twist angles, TMD interfaces also exhibit superlubric behavior.^26,27^

Notably, many of these highly complex phenomena can be rationalized in simple geometric terms.^28−37^ Specifically, the registry index (RI) approach provides an intuitive and highly computationally efficient geometric measure of the global (GRI) or the spatially resolved local (LRI) interlayer registry of rigid layered material interfaces as they slide atop each other.^15,35,36,38−42^ As such, it serves as a compelling characterization tool for commensurability-related interfacial phenomena. Previously, the GRI approach was successfully applied to show that the sliding energy landscape of homogeneous and heterogeneous interfaces, such as graphene/graphene, *h*-BN/*h*-BN, MoS_2_/MoS_2_, graphene/carbon nanotubes, and graphene/*h*-BN, is dictated by the interlayer registry.^15,36,38−42^ Furthermore, the LRI was shown to be a powerful tool for unveiling the physical mechanism underlying circumferential faceting in multiwalled nanotubes,^41^ rationalizing unique interlayer electronic transport characteristics in twisted graphitic interfaces,^3^ and analyzing the structural characteristics of reconstructed moiré superlattices in twisted *h*-BN junctions and their relation to the ferroelectric properties of the system.^4^

In light of the recent experimental developments mentioned above, it is desirable to extend the applicability of the RI approach to the treatment of a variety of homogeneous and heterogeneous TMD interfaces. Because these materials share common intralayer and interlayer spatial arrangements, a universal definition can be presented. To that end, we consider a TMD interface consisting of two layers, marked as *a* and *b*, and their chemical compositions are marked by  and , where M^*a*/*b*^ = Mo or W and X^*a*/*b*^ = S or Se. To mimic the Pauli repulsions experienced by atom *i*, of type t_*i*_ (=M^*a*^ or X^*a*^), residing in layer *a* due to atom *n*, of type t_*n*_ (=M^*b*^ or X^*b*^), residing on its adjacent layer *b*, we associate with it a two-dimensional Gaussian function of the following form:
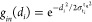
1where  represents the effective radius of atoms of type t_*i*_ in one layer when interacting with an atom of type t_*n*_ in its adjacent layer and *d*_*i*_ is the lateral distance from atom *i*. Next, the projected overlap, *s*_*in*_, between pairs of Gaussian functions associated with atoms *i* and *n*, residing in adjacent layers, is calculated via
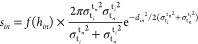
2where *d*_*in*_ is the lateral distance between the two atoms^42^ and  is a dimensionless scaling factor, used to describe the dependence of the repulsive interaction on vertical interatomic distance *h*_*in*_. Here, α is a fitting parameter and  is set to the interlayer (vertical) distance between atoms *i* and *n* at the optimal stacking mode. We note that in the present treatment we neglect all Gaussian overlaps with atoms residing in the external chalcogenide sublayers. With this, a universal TMD GRI expression may be defined as follows:
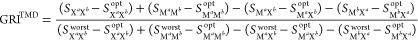
3where  is the sum of pairwise overlaps between all atoms of type T^*a*^ (=M^*a*^ or X^*a*^) in layer *a* with all atoms of type T^*b*^ (=M^*b*^ or X^*b*^) in layer *b* and  and  are the corresponding overlap sums obtained at the energetically worst and optimal stacking modes of the relevant interface, respectively. With this, the GRI^TMD^ expression is normalized to the range [0, 1], where 0 stands for the optimal interlayer registry and 1 represents the worst stacking mode in terms of the total energy.

The effective atomic radii appearing in the developed GRI^TMD^ expression are parametrized separately for each interface. To that end, we calculate the sliding energy landscape of the interface using state-of-the-art density functional theory (DFT) calculations (see section 1 of the Supporting Information for details). First, geometry optimization of the individual layers is performed. The relaxed layers are then stacked to form a bilayer model. For heterogeneous interfaces, the lateral lattice vectors of the bilayer supercell are chosen as the average of the lattice vectors of the relaxed individual layers in each direction, which are stretched or compressed accordingly. Finally, a set of single-point DFT calculations is performed on the bilayer system with the upper layer being rigidly shifted along the armchair direction (see Figure 1). The GRI^TMD^ effective radii are set to achieve optimal agreement between the reference DFT sliding energy landscape and the RI profile calculated for the same set of interlayer configurations (see section 2 of the Supporting Information for details regarding the fitting procedure). Structural parameters of the various bilayer systems considered in this study are provided in the Supporting Information section 1.

**Figure 1:**
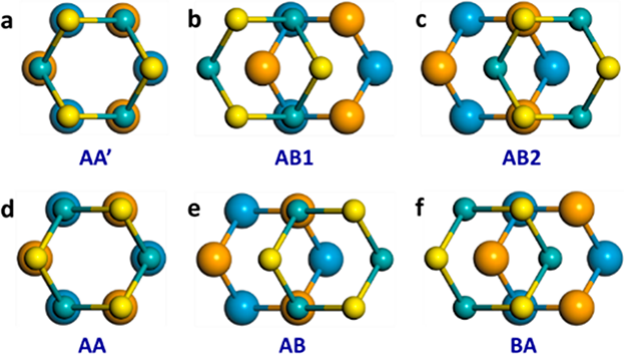
High-symmetry stacking modes of a (heterogeneous) TMD bilayer. The top panels represent a rigid shift among the (a) AA′, (b) AB1, and (c) AB2 stacking modes along the armchair direction of an antiparallel stacked bilayer. The bottom panels represent a rigid shift among the (d) AA, (e) AB, and (f) BA stacking modes along the armchair direction of a parallel stacked bilayer. For the sake of clarity, each atom type is given a different color, where the top (bottom) layer atoms are represented by small (large) spheres.

To evaluate the performance of the developed registry index approach for TMDs, we demonstrate it first for the case of the homogeneous WSe_2_ bilayer interface. Figure 2a compares the reference rigid shift  DFT sliding energy curves for parallel (full red line) and antiparallel (full blue line) stacked WSe_2_ bilayers with the corresponding GRI^TMD^ sliding profiles (dashed curves). The RI effective radii are fitted separately for the parallel and antiparallel stacking modes yielding the following values: , , , , , and , where , *t* = 3.287 Å is the lattice constant, and we choose  to reduce the number of free model parameters. Excellent agreement is found between the reference data and the GRI^TMD^ results, signifying the fact that the sliding energy corrugation is dictated by the interlayer lattice registry. We note that using a single parameter set for the parallel and antiparallel configurations provides satisfactory agreement between the GRI^TMD^ profiles and the DFT reference data for most of the systems considered with some deviations that are eliminated when using separate parameter sets (see Figure S10 and the corresponding discussion in section 2 of the Supporting Information).

**Figure 2:**
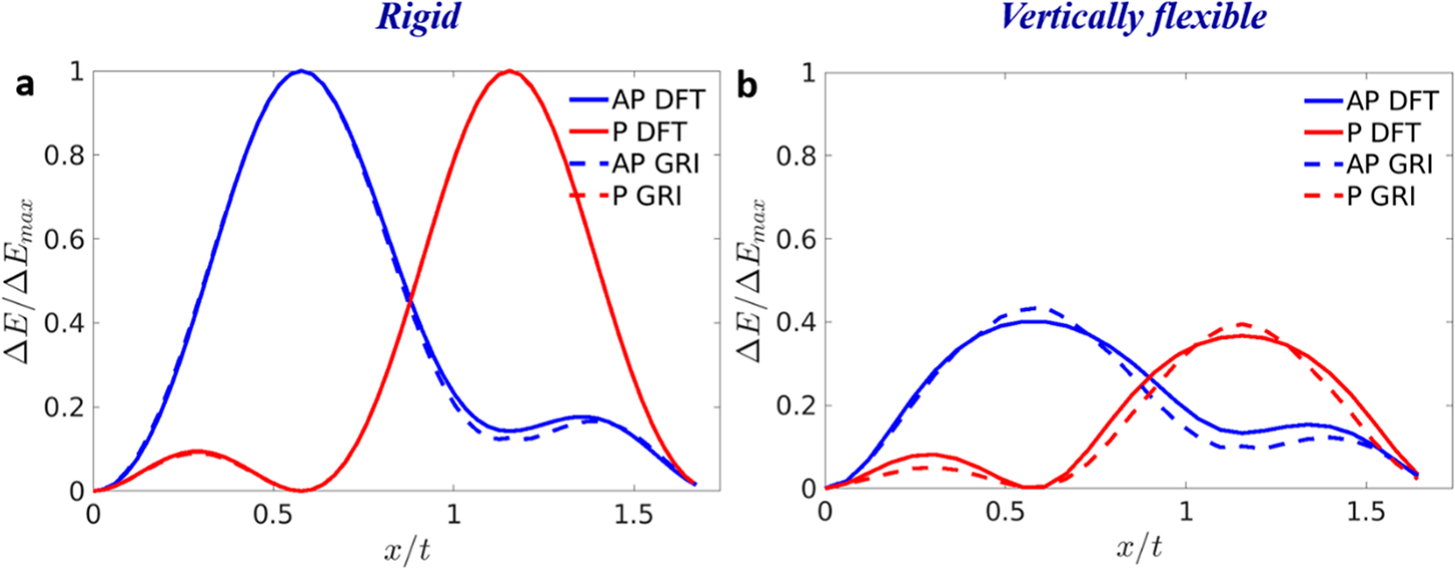
Sliding energy curves of the antiparallel (blue) and parallel (red) stacked homogeneous WSe_2_ bilayer calculated using density functional theory (full curves) and the GRI^TMD^ approach (dashed curves) for (a) rigid and (b) vertically relaxed shifts along the armchair direction. The origin of the DFT energy scale for the antiparallel and parallel configurations is set to the total energy of the AA′ (*E*_AA′_) and AB (*E*_AB_) stacking modes, and the curves are normalized by the energy of the rigidly shifted AB2  and AA  stacking modes, respectively. The initial AB stacking mode in the parallel configuration is constructed from the optimized AA′ stacking mode with a 60° rigid rotation of the top layer. Here, separate GRI^TMD^ parametrizations are used for the AP and P interlayer orientations. Note that the dashed red line in panel a resides atop the full red line and hence is difficult to notice.

The GRI^TMD^ can be further extended to capture structural relaxation effects during sliding. To demonstrate this, we compare in Figure 2b the DFT sliding curve along the same direction, where the vertical coordinates of all model atoms are allowed to relax following each shift, with the corresponding GRI^TMD^ profile. The latter was obtained using the same effective atomic radii while fitting the exponent α(AP) = 1.29 and α(P) = 1.06 (see eq 2) to obtain optimal agreement with the reference data. In this case, as well, the reference sliding energy curve incorporating complex interlayer electronic interaction effects can be described well by the simple GRI^TMD^ geometric measure. Note that the maximal GRI^TMD^ value obtained in this case is ∼0.4. This results from the fact that the GRI^TMD^ is normalized according to the rigidly shifted AA′ (optimal) and AB2 (worst) stacking configurations for antiparallel stackings and AB (optimal) and AA (worst) stacking configurations for parallel stackings. Because the sliding energy profiles of the rigidly- and vertically relaxed shifted bilayer systems have the same shape, the peak GRI value ratio of 0.4:1 is sufficient to reconstruct the entire profile of the vertically relaxed system from that of the rigidly shifted one (see section 3 of the Supporting Information).

The GRI^TMD^ parametrization mentioned above was fitted against one-dimensional sliding energy reference data. To verify its validity to represent sliding in directions other than the armchair direction, we test the GRI^TMD^ landscape obtained with the same parametrization against the entire rigidly shifted two-dimensional DFT sliding energy landscape (see Figure 3). Here, as well, good agreement between the reference DFT results and the GRI^TMD^ predictions is obtained, further supporting the validity of the approach.

**Figure 3:**
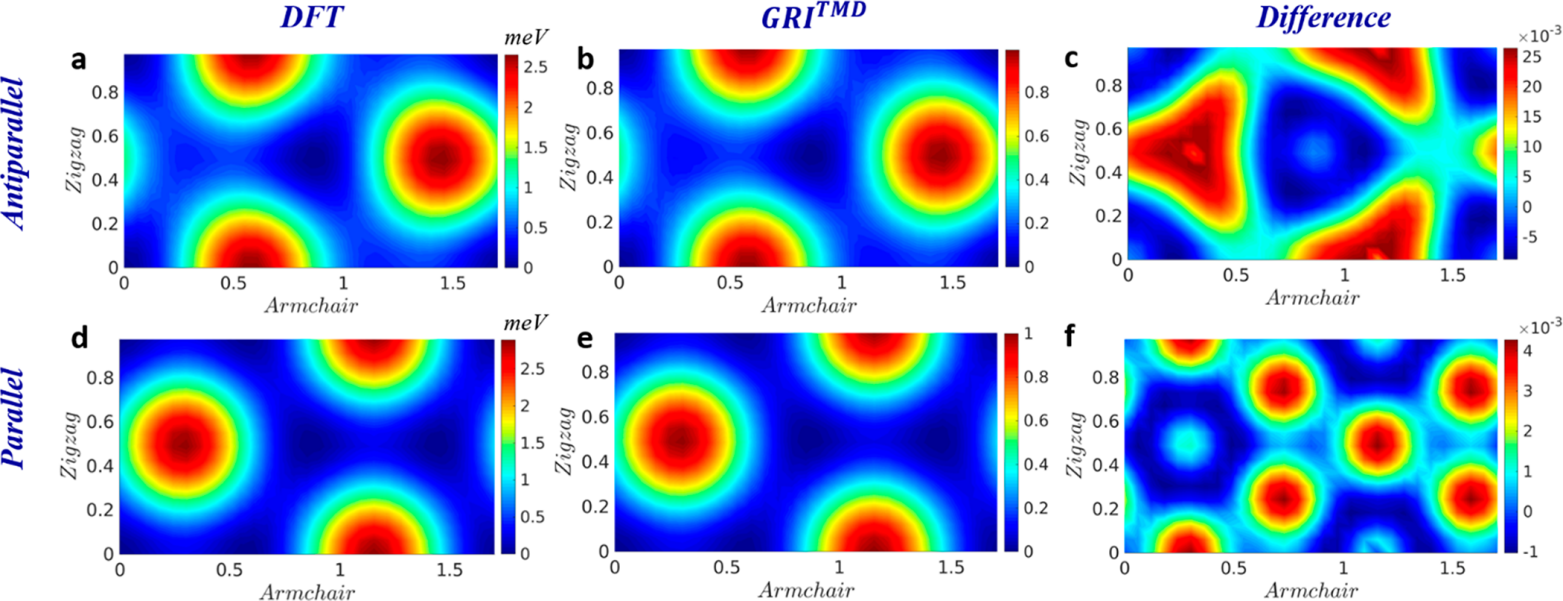
Two-dimensional potential energy surfaces (PESs) of bilayer WSe_2_ stacked in the antiparallel (top) or parallel (bottom) orientations calculated using DFT (left column) and the GRI^TMD^ (middle column) under rigid shifts along the armchair and zigzag directions. The energy origins of the DFT PESs obtained for the AP and P orientations are set to the total energies calculated at the same level of theory for the optimal AA′ and AB stacking modes, respectively, and the PESs are normalized by the energy of the worst AB2 and AA stacking modes for AP and P interlayer orientations (measured with respect to the corresponding origins), respectively. The right panels show the difference between the normalized DFT PESs and the corresponding GRI^TMD^ landscapes. In all panels, the *x*- and *y*-axis shifts are normalized with respect to the lattice constant of WSe_2_ (*t* = 3.287 Å). Color bars appear to the right of each panel.

Further information can be gained by defining the local registry index,^41^ LRI^TMD^, that provides a spatially resolved map of the degree of commensurability between the interfacing surfaces. To this end, we define the LRI^TMD^(i) at atomic position i in the top layer by applying Eq. 3 for atom i and each of its three nearest neighbors, p (in the adjacent internal sublayer of the same TMD layer), and averaging over the results obtained for the three i-p pairs. In practice, this is done by calculating the GRI^TMD^ while ignoring all atoms in the top layer apart from atom i and one of its three neighbors in the adjacent internal sublayer of the same TMD layer, p_1_. Then repeating this calculation for the i-p_2_ and i-p_3_ pairs and setting the LRI^TMD^(i) as the average of the three results.

To demonstrate the capabilities of the LRI measure, we consider the case of surface reconstruction in twisted MoS_2_ interfaces, which have been studied experimentally.^25^Figure 4 presents LRI^TMD^ maps for antiparallel (top panels) and parallel (bottom panel) stacked twisted bilayer MoS_2_ before (panels a and c) and after (panels b and d) geometry relaxation using an anisotropic MoS_2_ interlayer potential^43^ (see details regarding the geometry relaxation calculations in section 4 of the Supporting Information and LRI^TMD^ parameters for MoS_2_ in section 2 of the Supporting Information). The twist angles are chosen as 0.65° and 0.25° for the parallel and antiparallel orientations, respectively, in accordance with the experimental observations.^25^ The LRI^TMD^ maps clearly demonstrate the effects of stacking configuration and geometry relaxation on the moiré patterns. The good correspondence between the LRI^TMD^ maps of the relaxed MoS_2_ structures and experimentally measured domain wall width (see section 4 of the Supporting Information for LRI^TMD^ profiles across the domain wall) of MoS_2_ bilayers^25^ indicates the suitability of the LRI^TMD^ to analyze the structural properties of complex TMD interfaces.

**Figure 4:**
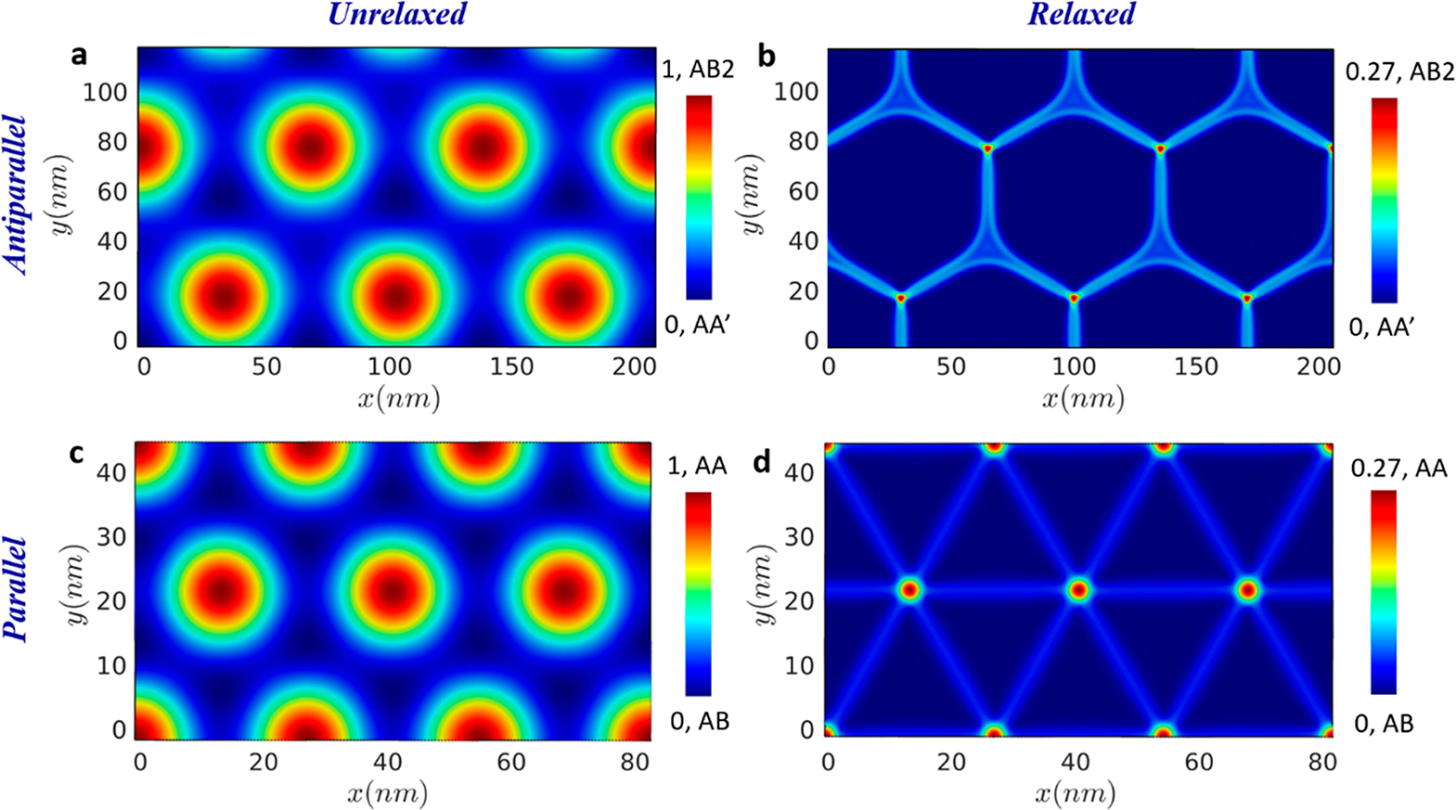
Local registry index calculated for (a and b) a 0.25° twisted antiparallel AA′ stacked and (c and d) a 0.65° twisted parallel AB stacked MoS_2_ bilayer before (left column) and after (right column) geometry relaxation using a dedicated interlayer potential (ILP).^43^ The moiré structure side lengths are 41.8 and 16.1 nm for the antiparallel and parallel twisted structures, respectively. Note that the LRI^TMD^ corrugation of the relaxed structures is somewhat lower than the maximal value obtained when using PBE+D3 DFT-based coordinates (see section 1 of the Supporting Information). This results from the fact that the ILP was parametrized against nonlocal many-body dispersion-corrected Heyd–Scuseria–Ernzerhof calculations, which produce a somewhat larger interlayer distance (see section 4 of the Supporting Information).

As stated above, the GRI^TMD^ approach is not limited to the case of homogeneous WSe_2_ interfaces. In section 2 of the Supporting Information, we provide a similar analysis for the homojunctions of MoS_2_, MoSe_2_, and WS_2_, demonstrating that once parametrized, the GRI^TMD^ captures well the sliding energy landscape of these systems, at a fraction of the computational cost of DFT calculations. The corresponding Gaussian width parameters are provided in section 2 of the Supporting Information. Here, we note that our present GRI^TMD^ expression includes metal–metal overlaps, which were previously neglected in the GRI parametrization of bilayer MoS_2_.^39^ Both approaches yield a good description of antiparallel orientation configurations (see section 5 of the Supporting Information), but the inclusion of metal–metal overlaps allows for an improved description of parallel orientation configurations, not considered previously in the context of the registry index.

The approach can be further extended to treat heterogeneous TMD bilayer structures. To demonstrate this, we consider the case of the WSe_2_/MoS_2_ bilayer, which has an inherent ∼4% interlayer lattice mismatch. As stated above, the GRI^TMD^ parametrization is performed against DFT reference data obtained for a stressed bilayer unit cell with a lattice parameter taken to be *t* = 3.221 Å - the average of the lattice constants of the relaxed individual layers, at the same level of theory (see section 1 of the Supporting Information for the calculation details). Figure 5 compares the DFT sliding energy curves (solid lines) obtained by rigid (a) and vertically flexible (b) interlayer shifts and the corresponding GRI^TMD^ curves (dashed lines) obtained for the antiparallel (blue) and parallel (red) interlayer orientations. Very good agreement between the GRI^TMD^ and the DFT reference data is obtained. Similar agreement is found for all other bilayer heterojunctions formed among MoS_2_, MoSe_2_, WS_2_, and WSe_2_ (see section 2 of the Supporting Information for a comprehensive analysis and the corresponding GRI^TMD^ parameters).

**Figure 5:**
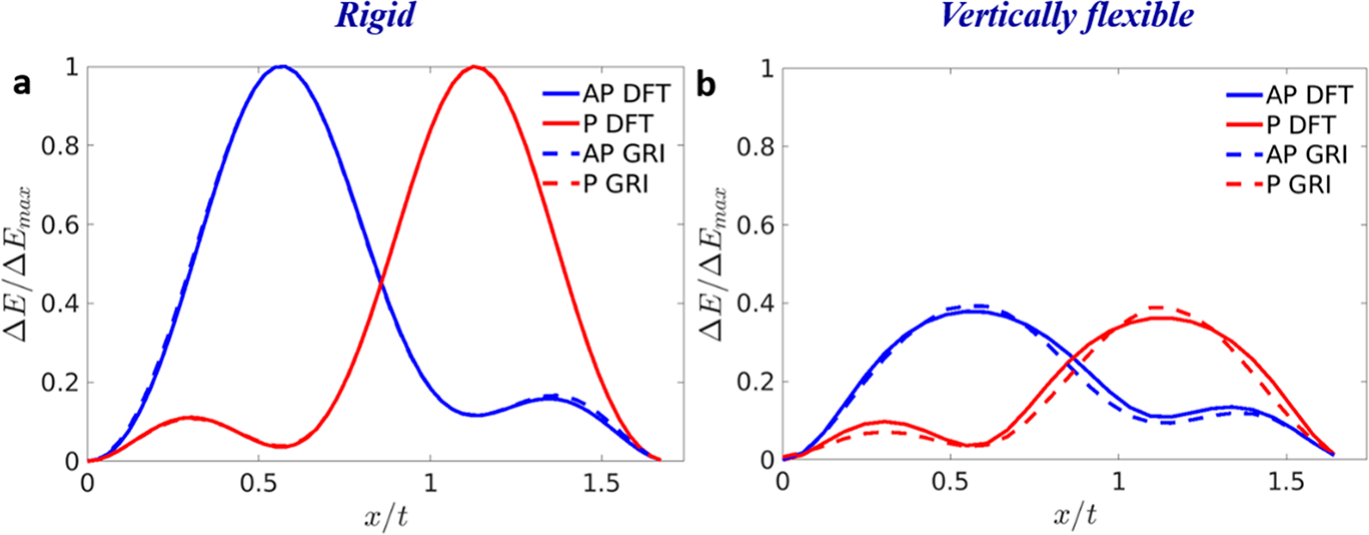
Sliding energy curves of the antiparallel (blue) and parallel (red) stacked WSe_2_/MoS_2_ heterogeneous bilayer calculated using density functional theory (full curves) and the GRI^TMD^ approach (dashed curves) for (a) rigid and (b) vertically flexible shifts along the armchair direction. The origin of the DFT energy scale for the antiparallel and parallel configurations is set to the total energy of the AA′ (*E*_AA′_) and AB (*E*_AB_) stacking modes, and the curves are normalized by the energy of the rigidly shifted AB2  and AA  stacking modes, respectively. The initial parallel AB stacking mode is built from the relaxed AA′ stacking mode with a 60° rotation of the top layer. Here, separate GRI^TMD^ parametrizations are used for the AP and P interlayer orientations.

The ability of the GRI^TMD^ to capture the rigid and vertically relaxed sliding potential energy surfaces of a variety of homogeneous and heterogeneous TMD interfaces indicates the versatility of our approach. A discussion of some possible limitations, however, is in place. Specifically, realistic material interfaces exhibit intrasurface elasticity effects that allow for the adjustment of the lateral positions of the slider atoms to the underlying potential. Hence, above a critical contact size that depends on the ratio between material elasticity and the stiffness of the interfacial interaction, locally commensurate regions may form, resulting in pinning effects and friction enhancement.^44−51^ In this respect, notable advantages of layered materials are their extremely stiff intralayer structure and relatively low interlayer sliding potential corrugation that may shift the critical length toward larger interface dimensions. Additionally, typical tribological scenarios involve contacts between three-dimensional objects, where interactions with the bulk support may suppress lateral elasticity effects in the contacting surfaces. Other complexities appearing in realistic frictional interfaces include surface roughness and contaminant adsorption, which may also lead to interfacial pinning.^33,52^ The former may actually break large-scale frictional interfaces into many nanoscale contacts, thus reducing undesirable elasticity effects.^10,53,54^ The latter can readily be remedied by standard running-in and annealing procedures.^17,55^ Therefore, together with the definition of the LRI^TMD^ that can characterize atomically reconstructed structures,^25^ the GRI^TMD^ approach provides a simple, intuitive, and highly computationally efficient approach for treating the structural, tribological, and even ferroelectric^4^ properties of complex TMD interfaces.

## Supporting Information Available

DFT methods, calculation of the global registry index, sliding energy curves without normalization, structural relaxation method, and registry index calculated by a previous definition (PDF)
Transparent Peer Review report available (PDF)
