# Supplementary material for: Interlayer Registry Index of Layered Transition Metal Dichalcogenides
Source: J Phys Chem Lett. 2022 Apr 8;13(15):3353–9. doi: 10.1021/acs.jpclett.1c04202 (PMC9140326; doi:10.1021/acs.jpclett.1c04202)
Supplement: Supplementary file 1 — jz1c04202_si_001.pdf [file jz1c04202_si_001.pdf]

Supporting Information

The Interlayer Registry Index of Layered Transition Metal Dichalcogenides

Wei Cao, Oded Hod, and Michael Urbakh*

Department of Physical Chemistry, School of Chemistry, The Raymond and Beverly Sackler Faculty of Exact Sciences and The Sackler Center for Computational Molecular and Materials Science, Tel Aviv University, Tel Aviv 6997801, Israel

*E-mail: odedhod@tauex.tau.ac.il

In this supplementary information, we provide additional details on certain aspects of the study reported in the manuscript. The following issues are discussed:

1. Density functional theory computational details.

2. Global registry index parameterization.

3. Unnormalized sliding energy curves.

4. Structural relaxation.

5. Comparison to previous MoS_2 registry index definition.

1. Density functional theory computational details

The total energy of the considered transition metal dichalcogenide (TMD) bilayers at various stacking modes was evaluated via density functional theory calculations using the plane-wave pseudopotential Quantum Espresso package.[1] The Perdew-Burke-Ernzerhof (PBE) generalized gradient exchange correlation functional approximation augmented by the Grimme-D3 dispersion correction with the Becke and Johnson (BJ) damping[2] and the PSlibrary[3] projector augmented wave (PAW) pseudopotentials were employed. This approach was shown to capture well the interlayer interactions in TMDs.[4] A plane wave energy cutoff of 60 Ry and a k-mesh of $12 \times 12 \times 1$ using the Monkhorst-pack scheme[5] were used for the calculation of the bilayer model. A sufficiently large vacuum size of 10 nm along the normal direction was set to avoid interactions between adjacent bilayer images. For systems exhibiting a finite electrostatic potential difference between the two layers, the dipole correction was applied.

Starting from the relaxed AA′ stacking mode of the antiparallel configurations, two types of calculations were performed: (i) rigid shift calculations, where the layers are laterally shifted rigidly with respect to each other along the armchair in steps of 0.1 Å and the total energy is calculated at each step; (ii) vertically flexible shift calculations, where at each step the vertical (z) coordinates of all atoms are allowed to relax prior to the total energy evaluation. The convergence threshold on forces for structural optimization was set to $1 \times 10^{-4} \frac{\mathrm{Ry}}{\mathrm{bohr}}$ and the relaxation was performed with a step size of 0.2 Å. These two simulation procedures were repeated for the parallel configuration, where the initial AB stacked bilayer was constructed from the relaxed AA′ configuration via a 60° rotation of the top layer.

Table S1 summarizes the structural parameters of the TMD homobilayer of MoS_2, $MoSe_2$, WS_2, and WSe_2, and the corresponding six heterobilayer models. Both the lattice constants (t) and the interlayer distances (h_0) of the structurally optimized AA′ stacking mode are provided. The interlayer distance is defined as the vertical distance between neighboring chalcogen atom in the adjacent layers. For the heterojunctions, a strained supercell was constructed with a lattice constant taken as the average of the lattice constants of the two isolated monolayers, relaxed at the same level of theory.

Table S1. DFT structural parameters of the various TMD bilayers at their AA' stacking modes.

System	t (Å)	h_0 (Å)
**Homobilayer**		
MoS_2	3.156	2.946
$MoSe_2$	3.284	3.084
WS_2	3.155	2.974
WSe_2	3.287	3.101
**Heterobilayer**		
MoS_2/WS_2	3.155	2.984
$MoSe_2/WSe_2$	3.286	3.078
$MoSe_2/MoS_2$	3.219	3.017
WSe_2/WS_2	3.222	3.031
$MoSe_2/WS_2$	3.219	3.005
WSe_2/MoS_2	3.221	3.014

For several high-symmetry stacking modes of the WSe_2 bilayer we performed reference calculation replacing the PBE functional approximation with the Heyd-Scuseria-Ernzerhof (HSE)[6-9] screened-exchange hybrid density functional. This functional, which is more computationally demanding, is known to be successful in describing the electronic properties of homogeneous and heterogeneous layered material structures.[10-16] As shown in Figure S1, the difference between the PBE and HSE calculated sliding energy barriers is within 0.6 meV/atom, marking the suitability of the PBE reference calculations for the GRI^{TMD} and LRI^{TMD} parameterization. Naturally, given a full reference HSE based dataset, one can readily reparametrize the L/GRI^{TMD}. This, however, is not expected to modify the qualitative nature of our results and conclusions.

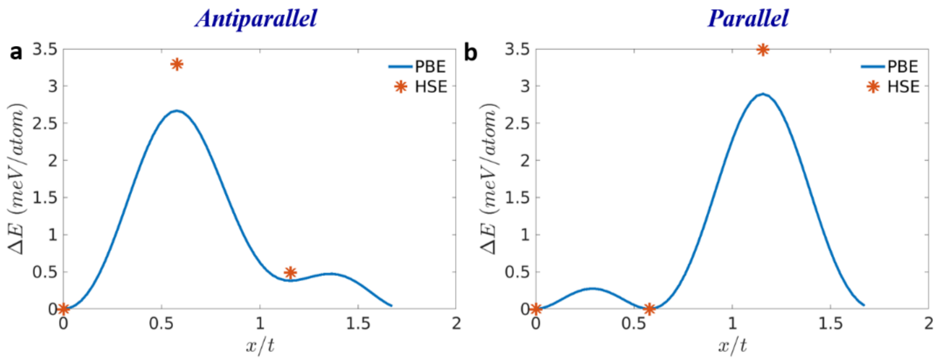

Figure S1. Sliding energy curves obtained by lateral rigid interlayer shifts of the antiparallel (a) and parallel (b) stacked homogeneous WSe_2 bilayer along the armchair direction. The full curves are PBE results, and asterisks are representative HSE energies. The origin of the energy scale for the anti-parallel and parallel configurations is set to the total energy of the AA′ and AB stacking modes.

2. Global registry index parameterization

The global registry index for transition metal dichalcogenides (GRI^{TMD}) was calculated via equations (1)-(3) in the main text. The effective atomic radii parameterization was performed against a set of ground state laterally periodic DFT calculations (see SI section 1 above) of the relevant bilayer at different interlayer stackings, obtained by a rigid relative interlayer shift along the armchair direction in steps of 0.1 Å. All atomic radii were scanned with a step of $0.01t/\sqrt{3}$ (t being the interlayer lattice constant of the relevant material), where for each set of radii the average difference between the GRI^{TMD} and normalized DFT predictions along the sliding path was recorded. The radii set producing the minimal average difference along the entire path was taken as the optimal solution. Table S2 and S3 present the corresponding effective atomic radii for various homogeneous and heterogeneous TMD interfaces, respectively. Similarly, the exponential parameter, α, was fitted against vertically flexible DFT interlayer shift calculations of the same bilayer system. For each interlayer configuration (using the same grid as in the rigid shift case) α was scanned in intervals of 0.01 $Å^{-1}$, and the GRI^{TMD} was evaluated using the optimal radii set. The value of α yielding the minimal average deviation from the vertically flexible DFT reference data was chosen as the optimal value. Table S4 lists the average and standard deviation differences between the reference DFT values and the GRI^{TMD} results obtained using the optimal parameter sets.

Table S2. Fitted GRI^{TMD} parameters for various homogeneous TMD bilayers. The effective radius of each atom pair appearing in the table is given in units of the $t/\sqrt{3}$, where *t* is the lattice constant (see table S1 above). P and AP stand for the parallel and anti-parallel interlayer orientations.

**MoS_2**	P	AP	**$MoSe_2$**	P	AP
σ_{Mo}^{Mo}	0.02	0.03	σ_{Mo}^{Mo}	0.01	0.02
σ_{W}^{S}	0.03	0.04	σ_{W}^{Se}	0.03	0.05
σ_{S}	0.3	0.3	σ_{Se}	0.3	0.3
α	1.19	1.34	α	1.14	1.32
**WS_2**	P	AP	**WSe_2**	P	AP
σ_{W}^{W}	0.01	0.02	σ_{W}^{W}	0.01	0.03
σ_{W}^{S}	0.03	0.05	σ_{W}^{Se}	0.02	0.05
σ_{S}	0.3	0.3	σ_{Se}	0.31	0.3
α	1.20	1.43	α	1.06	1.29

Table S3. Fitted GRI^{TMD} parameters for various heterogeneous TMD bilayers. The effective radius of each atom pair appearing in the table is given in units of $t/\sqrt{3}$, where t is the average lattice constant (see table S1 above) of the two monolayers. P and AP stand for the parallel and anti-parallel interlayer orientations.

**MoS_2/WS_2**	P	AP	**$MoSe_2/WSe_2$**	P	AP
σ_W^{Mo}	0.01	0.02	σ_W^{Mo}	0.01	0.03
σ_{Mo}^{W}	0.01	0.02	σ_{Mo}^{W}	0.01	0.03
σ_{Mo}^{S}	0.03	0.04	σ_{Mo}^{Se}	0.03	0.04
σ_W^{S}	0.03	0.05	σ_W^{Se}	0.03	0.06
σ_S	0.30	0.30	σ_{Se}	0.30	0.29
α	1.18	1.34	α	1.17	1.46
**$MoSe_2/MoS_2$**	P	AP	**WSe_2/WS_2**	P	AP
σ_{Mo}^{Mo}	0.01	0.03	σ_W^{W}	0.02	0.02
σ_{Mo}^{S}	0.04	0.04	σ_W^{S}	0.04	0.06
σ_{Mo}^{Se}	0.02	0.05	σ_W^{Se}	0.02	0.02
σ_S	0.31	0.27	σ_S	0.24	0.4
σ_{Se}	0.29	0.32	σ_{Se}	0.36	0.2
α	1.14	1.36	α	1.08	1.26
**$MoSe_2/WS_2$**	P	AP	**WSe_2/MoS_2**	P	AP
σ_W^{Mo}	0.02	0.03	σ_W^{Mo}	0.01	0.01
σ_{Mo}^{W}	0.02	0.03	σ_{Mo}^{W}	0.01	0.01
σ_{Mo}^{S}	0.04	0.03	σ_W^{S}	0.04	0.05
σ_W^{Se}	0.02	0.06	σ_{Mo}^{Se}	0.01	0.05
σ_S	0.33	0.26	σ_S	0.33	0.30
σ_{Se}	0.28	0.34	σ_{Se}	0.27	0.30
α	1.18	1.37	α	1.15	1.36

Table S4. Average ($\bar{x}$) difference between the reference DFT sliding energy profiles and the GRI^{TMD} results and the corresponding standard deviation (σ) obtained using the optimal parameter sets for each system.

	Rigid shift		Flexible shift			Rigid shift		Flexible shift	
**MoS_2**	P	AP	P	AP	**$MoSe_2$**	P	AP	P	AP
$\bar{x}$	0.0007	0.0038	0.0193	0.0182	$\bar{x}$	0.0006	0.0051	0.0187	0.0128
σ	0.0008	0.0028	0.0129	0.0122	σ	0.0005	0.0047	0.0133	0.0105
**WS_2**	P	AP	P	AP	**WSe_2**	P	AP	P	AP
$\bar{x}$	0.0008	0.0054	0.0180	0.0106	$\bar{x}$	0.0011	0.0098	0.0220	0.0204
σ	0.0006	0.0048	0.0137	0.0092	σ	0.0012	0.0088	0.0152	0.0165
**MoS_2/WS_2**	P	AP	P	AP	**$MoSe_2/WSe_2$**	P	AP	P	AP
$\bar{x}$	0.0011	0.0041	0.0180	0.0153	$\bar{x}$	0.0013	0.0058	0.0182	0.0107
σ	0.0015	0.0029	0.0126	0.0114	σ	0.0016	0.0044	0.0122	0.0093
**$MoSe_2/MoS_2$**	P	AP	P	AP	**WSe_2/WS_2**	P	AP	P	AP
$\bar{x}$	0.0010	0.0042	0.0184	0.0137	$\bar{x}$	0.0073	0.0032	0.0295	0.0192
σ	0.0012	0.0032	0.0123	0.0101	σ	0.0062	0.0029	0.0133	0.0115
**$MoSe_2/WS_2$**	P	AP	P	AP	**WSe_2/MoS_2**	P	AP	P	AP
$\bar{x}$	0.0024	0.0030	0.0197	0.0154	$\bar{x}$	0.0009	0.0047	0.0190	0.0119
σ	0.0021	0.0026	0.0109	0.0089	σ	0.0009	0.0046	0.0123	0.0095

In Figures S2-S9 we present the reference DFT sliding energy curves and the corresponding optimal GRI^{TMD} profiles for all bilayer models considered. The origin of the DFT energy scale for the anti-parallel (AP) and parallel (P) configurations is set to the total energy of the AA' ($E_{AA'}$) and AB (E_{AB}) stacking modes and the curves are normalized by the energy of the rigidly shifted $AB2$ ($\Delta E_{max}^{AP} = E_{AB2} - E_{AA'}$) and AA ($\Delta E_{max}^{P} = E_{AA} - E_{AB}$) stacking modes, respectively. The initial AB stacking mode is built from the relaxed AA' stacking mode via a 60° rotation of the top layer. Note that whenever the dashed line is invisible, it coincides with the full line of the same color.

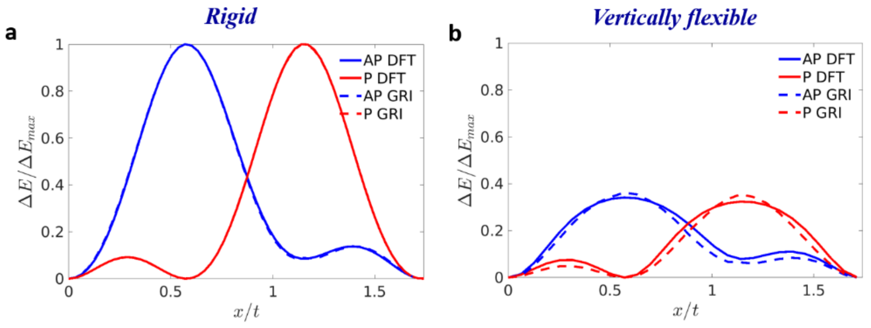

Figure S2. Sliding energy curves of the antiparallel (blue) and parallel (red) stacked homogeneous MoS_2 bilayer calculated using density functional theory (full curves) and the GRI^{TMD} approach (dashed curves) for (a) rigid and (b) vertically relaxed shifts along the armchair direction.

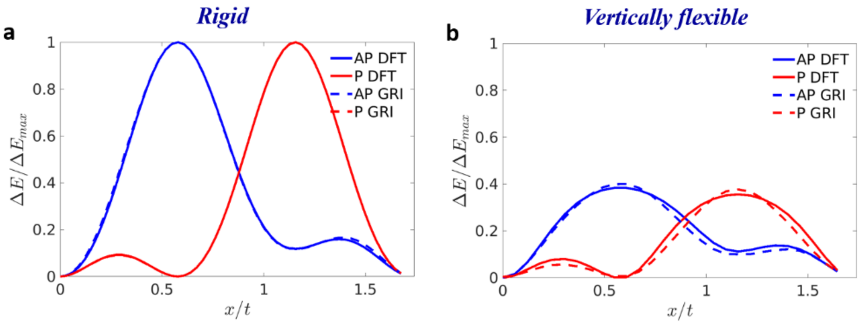

Figure S3. Sliding energy curves of the antiparallel (blue) and parallel (red) stacked homogeneous $MoSe_2$ bilayer calculated using density functional theory (full curves) and the GRI^{TMD} approach (dashed curves) for (a) rigid and (b) vertically flexible shifts along the armchair direction.

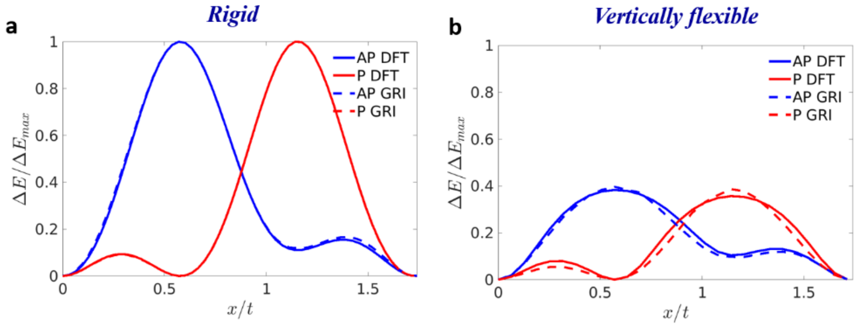

Figure S4. Sliding energy curves of the antiparallel (blue) and parallel (red) stacked homogeneous WS_2 bilayer calculated using density functional theory (full curves) and the GRI^{TMD} approach (dashed curves) for (a) rigid and (b) vertically flexible shifts along the armchair direction.

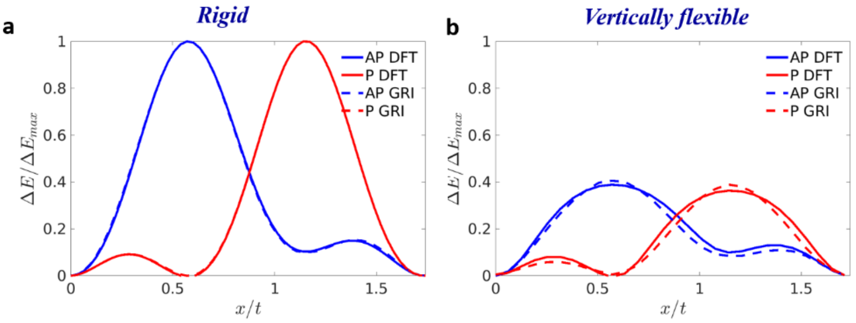

Figure S5. Sliding energy curves of the antiparallel (blue) and parallel (red) stacked MoS_2/WS_2 heterogeneous bilayer calculated using density functional theory (full curves) and the GRI^{TMD} approach (dashed curves) for (a) rigid and (b) vertically flexible shifts along the armchair direction.

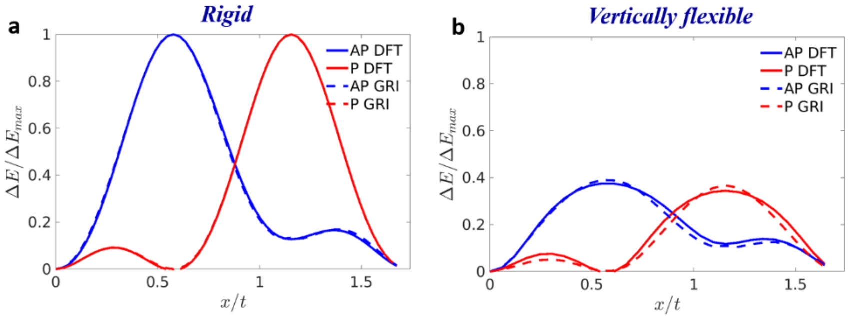

Figure S6. Sliding energy curves of the antiparallel (blue) and parallel (red) stacked $MoSe_2/WSe_2$ heterogeneous bilayer calculated using density functional theory (full curves) and the GRI^{TMD} approach (dashed curves) for (a) rigid and (b) vertically flexible shifts along the armchair direction.

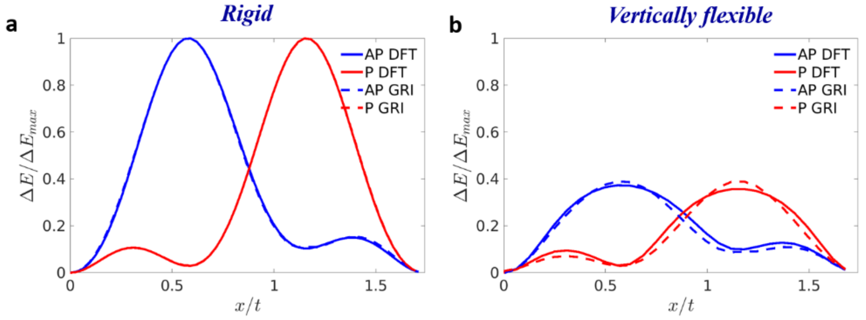

Figure S7. Sliding energy curves of the antiparallel (blue) and parallel (red) stacked $MoSe_2/MoS_2$ heterogeneous bilayer calculated using density functional theory (full curves) and the GRI^{TMD} approach (dashed curves) for (a) rigid and (b) vertically flexible shifts along the armchair direction.

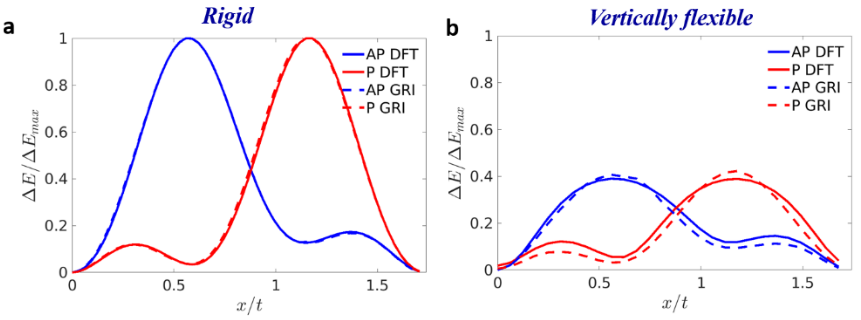

Figure S8. Sliding energy curves of the antiparallel (blue) and parallel (red) stacked WSe_2/WS_2 heterogeneous bilayer calculated using density functional theory (full curves) and the GRI^{TMD} approach (dashed curves) for (a) rigid and (b) vertically flexible shifts along the armchair direction.

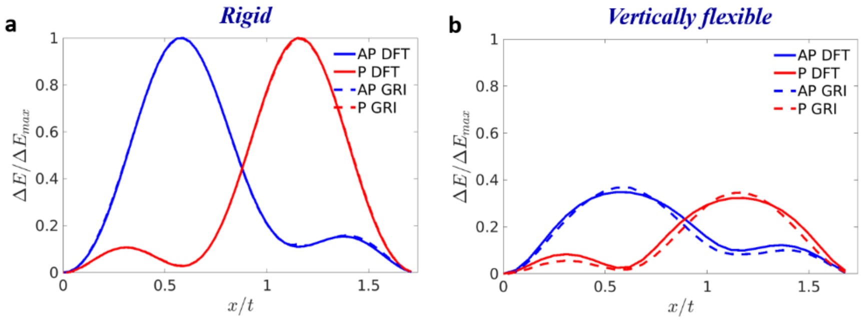

Figure S9. Sliding energy curves of the antiparallel (blue) and parallel (red) stacked $MoSe_2/WS_2$ heterogeneous bilayer calculated using density functional theory (full curves) and the GRI^{TMD} approach (dashed curves) for (a) rigid and (b) vertically flexible shifts along the armchair direction.

In the calculations presented above and in the main text we used separate GRI^{TMD} parameterizations for the AP and P interlayer orientations. Figure S10 demonstrates that one can obtain quite good agreement between the DFT reference data and the GRI^{TMD} results (with some minor deviations near $x = 1.2t$) for rigid shifts, even with a single parameter set. Somewhat larger deviations, however, appear for vertically flexible shifts, possibly due to variations in the interfacial electron density between the P and AP configurations. These, in turn, require different effective atomic radii to represent the corresponding Pauli repulsions.

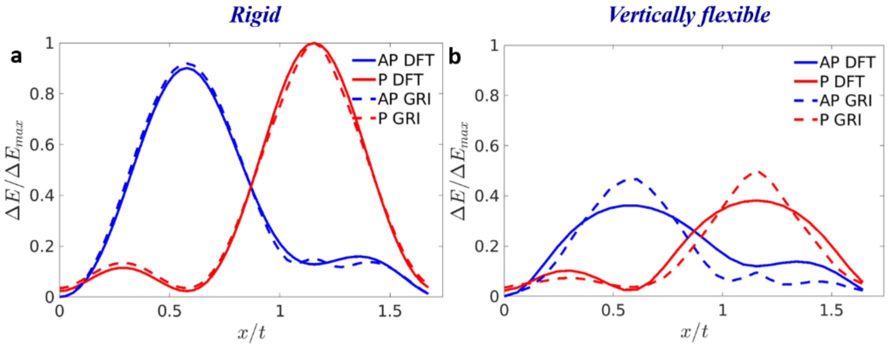

Figure S10. Sliding energy curves for the AP (blue) and P (red) stacked homogeneous WSe_2 bilayer calculated using DFT (full curves) and the GRI^{TMD} approach (dashed curves) for (a) rigid and (b) vertically relaxed shifts along the armchair direction. The GRI^{TMD} profiles are calculated using the following parameters for both of the AP and P configurations: $\sigma_W^W = 0.08b$, $\sigma_W^{Se} = 0.04b$, $\sigma_{\mathrm{Se}} = 0.4b$, $\alpha = 0.42$, where $b = t/\sqrt{3}$, $t = 3.287$ Å is the lattice constant.

3. Unnormalized sliding energy curves

As shown in SI section 2 above, the sliding energy profiles of all bilayers considered show very similar features and nearly overlap when presented in normalized form. For completeness, in Figures S11 and S12 we present the reference DFT sliding energy curves without normalization for the various homogeneous and heterogeneous bilayer systems considered in this work, respectively.

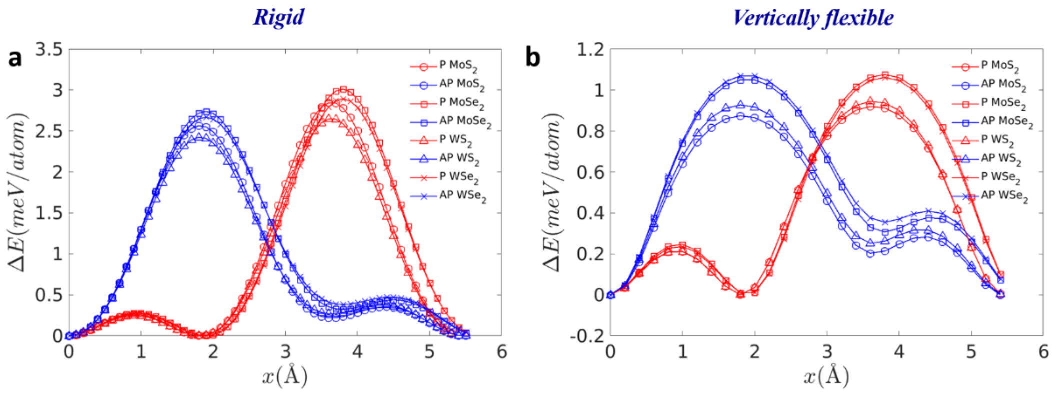

Figure S11. DFT sliding energy curves of the antiparallel (blue) and parallel (red) stacked homojunctions for (a) rigid and (b) vertically flexible shifts along the armchair direction. The origin of the energy scale for the anti-parallel and parallel configurations is set to the total energy of the AA' and AB stacking modes, respectively.

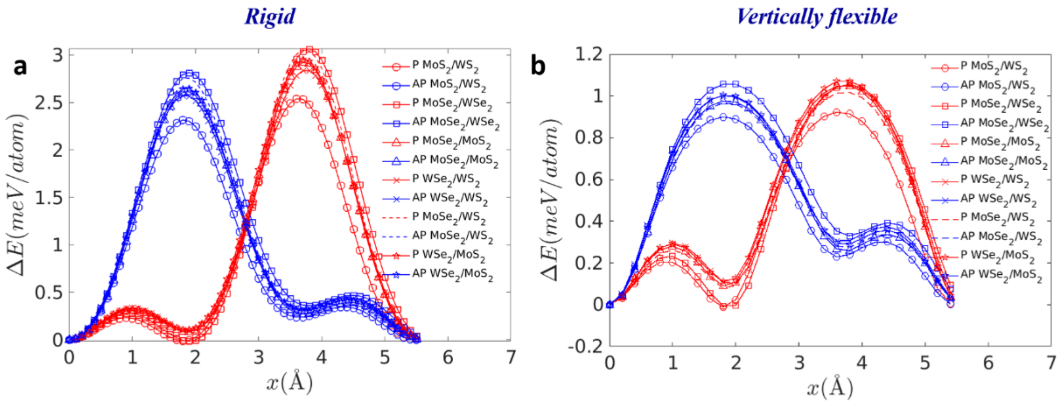

Figure S12. DFT sliding energy curves of the antiparallel (blue) and parallel (red) stacked heterojunctions for (a) rigid and (b) vertically flexible shifts along the armchair direction. The origin of the energy scale for the anti-parallel and parallel configurations is set to the total energy of the AA' and AB stacking modes, respectively.

4. Structural relaxation

In the main text, we presented a local registry index (LRI^{TMD}) analysis of surface reconstruction in twisted MoS_2 bilayers. To this end, supercells consisting of 7 full moiré patterns of twisted MoS_2 bilayers with $\theta = 0.25°$ for the antiparallel orientation and $\theta = 0.65°$ for the parallel orientation, were generated using the LAMMPS package.[17] Periodic boundary condition were applied in the lateral directions. In the vertical direction a sufficiently large vacuum size of 10 nm was applied to avoid spurious interactions between adjacent bilayer images. The intralayer interaction was described via the second-generation reactive empirical bond order (REBO) potential,[18] and the interlayer interaction was calculated by a dedicated registry dependent interlayer potential (ILP).[19] The Fire algorithm with a force tolerance of $10^{-6}\ eV/Å$ was used to relax the structure (including the box size) keeping the lower sulfur atomic sublayer of the bottom MoS_2 layer fixed to mimic a rigid substrate.

The LRI^{TMD} maps presented in Figure 4 of the main text conform well with the lattice domains found in experiments.[20] Specifically, when plotting a one-dimensional LRI^{TMD} profile cross section along a domain wall (see Figure S13) a typical width of ~ 4 nm (~5 nm) is found for the parallel (antiparallel) orientation in good agreement with measured values.[20]

Note that the LRI^{TMD} corrugation calculated for the ILP[19] relaxed MoS_2 bilayer is somewhat smaller than the GRI^{TMD} sliding profile presented in Fig. S2b above (e.g. ~0.27 versus ~0.4 for the AA stacking mode). This is mainly attributed to the fact that the ILP was parametrized against non-local many-body dispersion corrected Heyd-Scuseria-Ernzerhof calculations,[19] which produce a somewhat larger interlayer distance. For example, for the AB stacked MoS_2 bilayer, the HSE+MBD-NL interlayer distance is $\sim$0.2 Å larger than that obtained using PBE-D3.

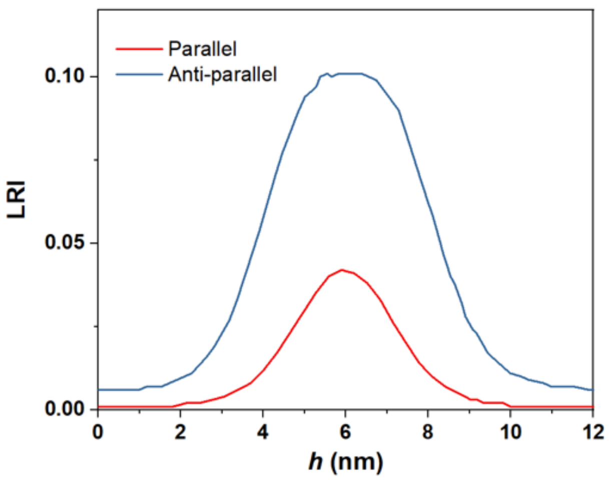

Figure S13. One-dimensional LRI^{TMD} profile cross section across domain walls in MoS_2 bilayers twisted by $\theta = 0.25°$ for the antiparallel (blue) and $\theta = 0.65°$ for the parallel (red) orientations.

5. Comparison to previous MoS_2 registry index definition

A GRI for MoS_2 was previously defined and parametrized against a local density approximation DFT sliding energy landscape obtained under external pressures of 500 MPa and 15 GPa.[21-22] This parameterization, which considered only the antiparallel interlayer configuration, provided good agreement with the reference data without accounting for Mo-Mo interactions. For completeness, we compare the GRI profile obtained using the previous definition and parameterization with our new DFT reference data for vertically flexible shifts at the antiparallel configuration. The results, appearing in Figure S14, show good agreement between the previous GRI definition and the new reference DFT data with the former presenting more sharp features due to the use of sharp circles (rather than smooth Gaussian) to represent effective atomic volumes. We note however, that the previous definition is insufficient to provide a good description of the sliding energy profile of the parallel configuration, hence the need for the new extended definition.

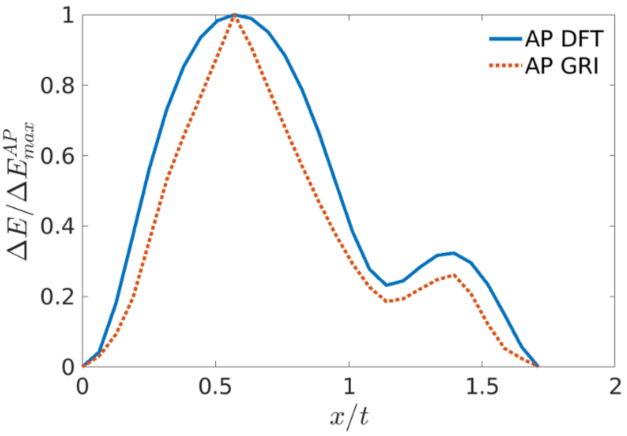

Figure S14. Sliding energy curves of the antiparallel stacked homogeneous MoS_2 bilayer calculated for vertically flexible shifts along the armchair direction using density functional theory (full curves) and the GRI^{TMD} approach (dashed curves) using the previous definition.[23] The origin of the DFT energy scale is set to the total energy of the AA' ($E_{AA'}$) stacking mode and the curve is normalized by the energy of the $AB2$ ($\Delta E_{max}^{AP} = E_{AB2} - E_{AA'}$) stacking mode.

References:

1). Giannozzi, P.; Baroni, S.; Bonini, N.; Calandra, M.; Car, R.; Cavazzoni, C.; Ceresoli, D.; Chiarotti, G. L.; Cococcioni, M.; Dabo, I.; Dal Corso, A.; de Gironcoli, S.; Fabris, S.; Fratesi, G.; Gebauer, R.; Gerstmann, U.; Gougoussis, C.; Kokalj, A.; Lazzeri, M.; Martin-Samos, L.; Marzari, N.; Mauri, F.; Mazzarello, R.; Paolini, S.; Pasquarello, A.; Paulatto, L.; Sbraccia, C.; Scandolo, S.; Sclauzero, G.; Seitsonen, A. P.; Smogunov, A.; Umari, P.; Wentzcovitch, R. M. QUANTUM ESPRESSO: a modular and open-source software project for quantum simulations of materials. *J. Phys.: Condens. Matter* **2009,** *21* (39), 395502.

(2). Grimme, S.; Antony, J.; Ehrlich, S.; Krieg, H. A consistent and accurate ab initio parametrization of density functional dispersion correction (DFT-D) for the 94 elements H-Pu. *J. Chem. Phys.* **2010,** *132* (15), 154104.

(3). Prandini, G.; Marrazzo, A.; Castelli, I. E.; Mounet, N.; Marzari, N. Precision and efficiency in solid-state pseudopotential calculations. *npj Comput. Mater.* **2018,** *4* (1), 72, 1-13.

(4). Wang, X.; Yasuda, K.; Zhang, Y.; Liu, S.; Watanabe, K.; Taniguchi, T.; Hone, J.; Fu, L.; Jarillo-Herrero, P. Interfacial ferroelectricity in rhombohedral-stacked bilayer transition metal dichalcogenides. *Nature Nanotechnology* **2022**, 1-5.

(5). Monkhorst, H. J.; Pack, J. D. Special points for Brillouin-zone integrations. *Phys. Rev. B* **1976,** *13* (12), 5188-5192.

(6). Heyd, J.; Scuseria, G. E.; Ernzerhof, M. Hybrid functionals based on a screened Coulomb potential. *J. Chem. Phys.* **2003,** *118* (18), 8207-8215.

(7). Heyd, J.; Scuseria, G. E. Assessment and validation of a screened Coulomb hybrid density functional. *J. Chem. Phys.* **2004,** *120* (16), 7274-7280.

(8). Heyd, J.; Scuseria, G. E. Efficient hybrid density functional calculations in solids: Assessment of the Heyd–Scuseria–Ernzerhof screened Coulomb hybrid functional. *The J. Chem. Phys.* **2004,** *121* (3), 1187-1192.

(9). Heyd, J.; Scuseria, G. E.; Ernzerhof, M. Erratum: "Hybrid functionals based on a screened Coulomb potential" [J. Chem. Phys. 118, 8207 (2003)]. *J. Chem. Phys.* **2006,** *124* (21), 219906.

(10). Marom, N.; Bernstein, J.; Garel, J.; Tkatchenko, A.; Joselevich, E.; Kronik, L.; Hod, O. Stacking and registry effects in layered materials: the case of hexagonal boron nitride. *Phys. Rev. Lett.* **2010,** *105* (4), 046801.

(11). Barone, V.; Hod, O.; Peralta, J. E.; Scuseria, G. E. Accurate prediction of the electronic properties of low-dimensional graphene derivatives using a screened hybrid density functional. *Acc. Chem. Res.* **2011,** *44* (4), 269-279.

(12). Barone, V.; Peralta, J. E. Magnetic boron nitride nanoribbons with tunable electronic properties. *Nano Lett.* **2008,** *8* (8), 2210-2214.

(13). Berseneva, N.; Krasheninnikov, A. V.; Nieminen, R. M. Mechanisms of postsynthesis doping of boron nitride nanostructures with carbon from first-principles simulations. *Phys. Rev. Lett.* **2011,** *107* (3), 035501.

(14). Zhu, J.; Bhandary, S.; Sanyal, B.; Ottosson, H. Interpolation of atomically thin hexagonal boron nitride and graphene: electronic structure and thermodynamic stability in terms of all-carbon conjugated paths and aromatic hexagons. *J. Phys. Chem. C* **2011,** *115* (20), 10264-10271.

(15). Liu, Y.; Wu, X.; Zhao, Y.; Zeng, X. C.; Yang, J. Half-metallicity in hybrid graphene/boron nitride nanoribbons with dihydrogenated edges. *J. Phys. Chem. C* **2011,** *115* (19), 9442-9450.

(16). Du, A.; Sanvito, S.; Li, Z.; Wang, D.; Jiao, Y.; Liao, T.; Sun, Q.; Ng, Y. H.; Zhu, Z.; Amal, R.; Smith, S. C. Hybrid graphene and graphitic carbon nitride Nanocomposite:

gap opening, electron–hole puddle, interfacial charge transfer, and enhanced visible light response. *J. Am. Chem. Soc.* **2012,** *134* (9), 4393-4397.
(17). Plimpton, S. Fast parallel algorithms for short-range molecular dynamics. *J. Comput. Phys.* **1995,** *117* (1), 1-19.
(18). Liang, T.; Phillpot, S. R.; Sinnott, S. B. Erratum: Parametrization of a reactive many-body potential for Mo--S systems [Phys. Rev. B 79, 245110 (2009)]. *Phys. Rev. B* **2012,** *85* (19), 199903.
(19). Ouyang, W.; Sofer, R.; Gao, X.; Hermann, J.; Tkatchenko, A.; Kronik, L.; Urbakh, M.; Hod, O. Anisotropic interlayer force field for transition metal dichalcogenides: the case of molybdenum disulfide. *J. Chem. Theory Compu.t* **2021,** *17* (11), 7237-7245.
(20). Weston, A.; Zou, Y.; Enaldiev, V.; Summerfield, A.; Clark, N.; Zólyomi, V.; Graham, A.; Yelgel, C.; Magorrian, S.; Zhou, M.; Zultak, J.; Hopkinson, D.; Barinov, A.; Bointon, T. H.; Kretinin, A.; Wilson, N. R.; Beton, P. H.; Fal'ko, V. I.; Haigh, S. J.; Gorbachev, R. Atomic reconstruction in twisted bilayers of transition metal dichalcogenides. *Nat. Nanotechnol.* **2020,** *15* (7), 592-597.
(21). Liang, T.; Sawyer, W. G.; Perry, S. S.; Sinnott, S. B.; Phillpot, S. R. First-principles determination of static potential energy surfaces for atomic friction in MoS_2 and MoO_3. *Phys. Rev. B* **2008,** *77* (10), 104105.
(22). Cahangirov, S.; Ataca, C.; Topsakal, M.; Sahin, H.; Ciraci, S. Frictional figures of merit for single layered nanostructures. *Phys. Rev. Lett.* **2012,** *108* (12), 126103.
(23). Blumberg, A.; Keshet, U.; Zaltsman, I.; Hod, O. Interlayer registry to determine the sliding potential of layered metal dichalcogenides: the case of 2H-MoS_2. *J. Phys. Chem. Lett.* **2012,** *3* (15), 1936-1940.
