# Supplementary material for: Interlayer Registry Index of Layered Transition Metal Dichalcogenides
Source: J Phys Chem Lett. 2022 Apr 8;13(15):3353–9. doi: 10.1021/acs.jpclett.1c04202 (PMC9140326; doi:10.1021/acs.jpclett.1c04202)
Supplement: Supplementary file 2 — jz1c04202_si_002.pdf [file jz1c04202_si_002.pdf]

jz-2021-04202c.R1

Name: Peer Review Information for "The Interlayer Registry Index of Layered Transition Metal Dichalcogenides"

First Round of Reviewer Comments

Reviewer: 1

Comments to the Author

Manuscript Review ID : jz-2021-04202c

The manuscript by W. Cao, et al. “The interlayer registry index of layered transition metal dichalcogenides” deals with an interesting and useful extension of the intuitive registry index framework as a powerful computational tool to quantitatively evaluate the interlayer commensurability at homogeneous and heterogeneous interfaces of 2D layered materials.
Previously shown to capture the sliding energy landscape at graphitic and hexagonal boron nitride interfaces (with important mechanical and tribological implications), in this Letter the applicability of the registry index approach is specifically extended by the authors towards the treatment of a variety of transition metal dichalcogenides possessing a threefold sublayer structure (e.g., MoS2, WS2, etc.).
The manuscript reports a very good agreement between the parametrized registry index landscape and the sliding energy landscape calculated using density functional theory for both homogeneous and heterogeneous junctions of these materials. Furthermore, it is shown that this developed geometric measure is highly suitable for characterizing surface reconstruction in twisted transition metal dichalcogenides interfaces that dictates their intricate electronic properties.

The scientific originality, importance, and significant advance of this work is clear, and the paper is well written and self-contained.
Recommending certainly its publication in the Journal of Physical Chemistry Letters, I would suggest anyhow the authors to address/consider the following questions:

- How many fitting parameters are introduced considering the global registry index approach for the description of homogeneous and heterogeneous TMD interfaces?

- For the same pair of contacting TMD materials the registry index is parametrized separately for the parallel and anti-parallel stacking modes yielding different values of effective radii. What is the reason for this approach and how can it be physically justified? Would it be possible, under some specific conditions, to quantify the TMD contact energy landscape using the same RI parameterization for the parallel and anti-parallel stacking modes?

- Authors found that the corrugation of local registry index surfaces calculated using relaxed structures is lower than the corrugation obtained considering the global registry index. What is the origin of this difference and what differences in the friction forces calculated by the two methods can this effect lead to?

Reviewer: 2

Comments to the Author

Cao and coworkers report the usefulness of the registry index (RI) to rationalize the frictional properties of layered transition metal dichalcogenides (LTMDs).

The paper opens with a well written, interesting paragraph on the intriguing properties of LTMDs. The authors then go on to investigate if the RI, which is constructed to roughly approximate the repulsion between adjacent layers, correlates with the frictional or transverse forces between the layers. Of course, they do, since resistance to sliding mainly originates from the repulsion between atoms.

The positive aspect of the RI is that it is more quantitative than a Moire pattern and more intuitive than a full DFT simulation. This is the point that the authors intend to emphasize. But the real comparison to make is that the RI analysis is less accurate than a full DFT or force-field based simulation and less intuitive than a Moire pattern. And I feel that this is the comparison that needs be made, also because the RI approach needs atomic position as input, so that we need the complexity of a full simulation as input information and then we already know the forces.

In conclusion, the paper adds neither qualitative insight nor new ways to predict frictional forces without having to run elaborate simulations first. For this reason, I cannot recommend the manuscript for publication, although the presentation itself is excellent.

Reviewer: 3

Comments to the Author

The paper is technically impeccable and the model seems to provide an excellent interpolation of the DFT results. I am more concerned regarding publication in Journal of Physical Chemistry Letters. Obviously the idea is not novel, apart from certain technicalities, and I do not see its predictive power for structural properties, let alone for the electronic properties claimed in the introduction. There is only one example presented in the text (the case of a large moire) and it is not properly described what makes it very difficult to appreciate the real accuracy of the prediction.

Author's Response to Peer Review Comments:

Please find attached the cover letter and detailed response to the referee reports.

19.02.2022

Dear Prof. Editor,

We thank you for communicating with us the review reports on our manuscript (jz-2021-04202c) titled “The Interlayer Registry Index of Layered Transition Metal Dichalcogenides”, recently submitted for your consideration for publication in the *Journal of Physical Chemistry Letters*. We also want to express our sincere appreciation to the referees for providing valuable comments and suggestions that have helped us in improving our manuscript.

Attached to this cover letter, please find our point-by-point response to all the comments raised by the referees and their corresponding suggestions. Also, please find attached the revised manuscript and SI file, including the highlighted modifications.

We believe that our responses have adequately addressed the referees’ comments and we hope that you will share our view that our manuscript, in its revised form, merits publication in the *Journal of Physical Chemistry Letters*.

Sincerely yours,

Oded Hod,

School of Chemistry

Tel-Aviv University

6997801 Tel-Aviv, Israel

Phone: 972-3-640-5850

e-mail: odedhod@tau.ac.il

homepage: http://www.tau.ac.il/~odedhod/

Reviewer 1

"The manuscript by W. Cao, et al. "The interlayer registry index of layered transition metal dichalcogenides" deals with an interesting and useful extension of the intuitive registry index framework as a powerful computational tool to quantitatively evaluate the interlayer commensurability at homogeneous and heterogeneous interfaces of 2D layered materials. Previously shown to capture the sliding energy landscape at graphitic and hexagonal boron nitride interfaces (with important mechanical and tribological implications), in this Letter the applicability of the registry index approach is specifically extended by the authors towards the treatment of a variety of transition metal dichalcogenides possessing a threefold sublayer structure (e.g., MoS_2, WS_2, etc.).

The manuscript reports a very good agreement between the parametrized registry index landscape and the sliding energy landscape calculated using density functional theory for both homogeneous and heterogeneous junctions of these materials. Furthermore, it is shown that this developed geometric measure is highly suitable for characterizing surface reconstruction in twisted transition metal dichalcogenides interfaces that dictates their intricate electronic properties.

The scientific originality, importance, and significant advance of this work is clear, and the paper is well written and self-contained.

Recommending certainly its publication in the Journal of Physical Chemistry Letters, I would suggest anyhow the authors to address/consider the following questions:"

Response:

We thank the reviewer for the positive evaluation of our manuscript and for recommending it for publication.

"- How many fitting parameters are introduced considering the global registry index approach for the description of homogeneous and heterogeneous TMD interfaces?"

Response:

We thank the reviewer for raising this point.

For homogeneous TMD interfaces the global registry index definition includes three fitting parameters for the rigid shift sliding energy curve, and an additional single parameter to model the effect of interlayer distance in vertically flexible shift calculations. For example, the following 3 effective radii (see Eq. (1)) are used to fit the rigid shift DFT sliding energy curves separately for the parallel and anti-parallel stacking modes of bilayer WSe_2: $\sigma_{\mathrm{W}}^{\mathrm{W}}$, σ_{W}^{Se}, and σ_{Se}. To fit the vertically flexible shift DFT sliding energy landscape, the same effective radii values obtained for the rigid shift fitting are used while fitting the α parameter in Eq. (2).

For heterogeneous TMD interfaces more fitting parameters are needed. Specifically, for the MoS_2/WS_2, $MoSe_2/WSe_2$, $MoSe_2/MoS_2$, WSe_2/WS_2 bilayers five effective radii are required and for the $MoSe_2/WS_2$, WSe_2/MoS_2 bilayers we find it necessary to define six effective radii to obtain good agreement with the rigid shift DFT sliding energy surfaces. For example, for the WSe_2/MoS_2

bilayer the following effective radii (see Eq. (1)) are used to fit rigid DFT shift data $\sigma_{\mathrm{W}}^{\mathrm{Mo}}$, $\sigma_{\mathrm{Mo}}^{\mathrm{W}}$, $\sigma_{\mathrm{W}}^{\mathrm{S}}$, $\sigma_{\mathrm{Mo}}^{\mathrm{Se}}$, σ_{S}, σ_{S}. As in the case of homogeneous interfaces, an additional single parameter α is required to capture vertical flexibility effects.

We note that all fitting parameter values are summarized in Tables S2 and S3 of the Supplementary Material.

"- For the same pair of contacting TMD materials the registry index is parametrized separately for the parallel and anti-parallel stacking modes yielding different values of effective radii. What is the reason for this approach and how can it be physically justified? Would it be possible, under some specific conditions, to quantify the TMD contact energy landscape using the same RI parameterization for the parallel and anti-parallel stacking modes?"

Response:

We thank the reviewer for raising this delicate, yet important, point. In fact, in our original formulation we obtained a unified set of parameters for both parallel (P) and anti-parallel (AP) configurations. Indeed, the agreement between the RI and DFT calculated sliding energy landscapes was quite satisfactory for most of the systems considered even with this single parameter set. We note, however, that due to some delicate opposite overlap contributions, the GRI^{TMD} rigid sliding landscape of the AP configurations developed some minor local extrema that are lacking in the DFT reference curves. Hence, to obtain and even better agreement, we opted to present a separate parameter set (yet with the same GRI^{TMD} functional form) for the parallel and anti-parallel configurations. Furthermore, we found it also valuable to use two separate parameter sets when describing vertically flexible shifts.

For example, Figure R1 presents DFT sliding energy curves (full lines) for the WSe_2 bilayer compared to the corresponding GRI^{TMD} sliding profiles (dashed lines) obtained using the following parameters for both the AP (blue) and P (red) cases: $\sigma_W^W = 0.08b$, $\sigma_W^{Se} = 0.04b$, $\sigma_{\mathrm{Se}} = 0.4b$, $\alpha = 0.42$, where $b = t/\sqrt{3}$ and $t = 3.287$ Å is the lattice constant. This is to be compared with the following values used to obtain the results appearing in Fig. 2: $\sigma_W^W(AP) = 0.03b$, $\sigma_W^{Se}(AP) = 0.05b$, $\sigma_{\mathrm{Se}}(AP) = 0.3b$, $\sigma_W^W(P) = 0.01b$, $\sigma_W^{Se}(P) = 0.02b$, $\sigma_{\mathrm{Se}}(P) = 0.31b$, fitted separately for the AP and P configurations, $\alpha(AP) = 1.29$, $\alpha(P) = 1.06$ (note that while doing the revision, we found a misprint in the way the definitions of the effective radii were written in the manuscript, which are corrected in the revised form).

For rigid shift configurations (left panel), the reference sliding energy curves match well with the GRI^{TMD} profiles, with some minor oscillations appearing at $x/t \approx 1.2$ in the AP GRI^{TMD} profile, which are absent in the reference DFT curve. We note that using a smaller σ_W^W and a larger σ_{Se} would decrease these oscillation amplitudes. However, this would be on the expense of reducing the agreement with the reference data at $x/t \approx 0.6$.

For the case of vertically flexible shifts (right panel), along with the local maximum appearing in the AP GRI^{TMD} profile at $x/t \approx 1.2$, the overall agreement between the reference DFT data and the RI profile is reduced when using a single parameter set, and specifically a single α value.

This demonstrates the reasoning behind our choice to use separate parameter sets for the AP and P configurations.

To make this point clearer, we have added Fig. R1 to supplementary section 2 as Figure S10 with the following discussion: "In the calculations presented above and in the main text we used separate GRI^{TMD} parameterizations for the AP and P interlayer orientations. Figure S10 demonstrates that one can obtain quite good agreement between the DFT reference data and the GRI^{TMD} results (with some minor deviations near $x = 1.2t$) for rigid shifts, even with a single parameter set. Somewhat larger deviations, however, appear for vertically flexible shifts, possibly due to variations in the interfacial electron density between the P and AP configurations. These, in turn, require different effective atomic radii to represent the corresponding Pauli repulsions.". We further added the following reference on page 7 of the revised main text: "We note that using a single parameter set for both the parallel and anti-parallel configurations provides satisfactory agreement between the GRI^{TMD} profiles and the DFT reference data for most of the systems considered with some deviations that are eliminated when using separate parameter sets (see Fig. S10 in SI section 2 and the corresponding discussion therein)."

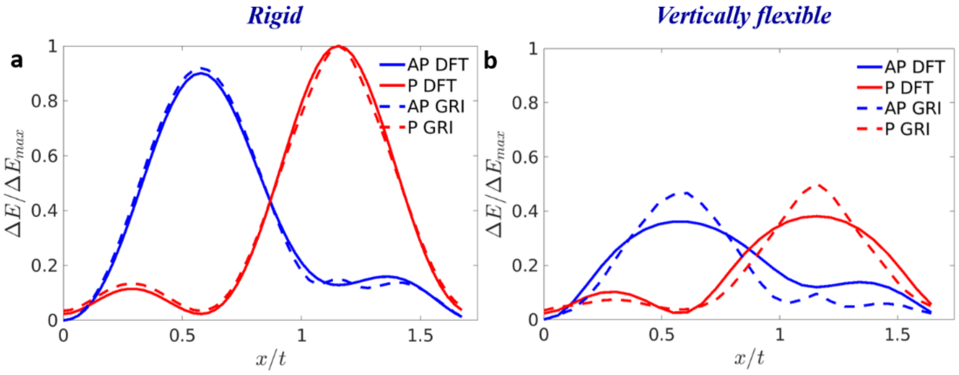

Figure R1. Sliding energy curves for the AP (blue) and P (red) stacked homogeneous WSe2 bilayer calculated using DFT (full curves) and the GRI^{TMD} approach (dashed curves) for (a) rigid and (b) vertically relaxed shifts along the armchair direction. The GRI^{TMD} profiles are calculated using the following parameters for both of the AP and P configurations: $\sigma_W^W = 0.08b$, $\sigma_W^{Se} = 0.04b$, $\sigma_{Se} = 0.4b$, $\alpha = 0.42$, where $b = t/\sqrt{3}$, $t = 3.287$ Å is the lattice constant.

"- Authors found that the corrugation of local registry index surfaces calculated using relaxed structures is lower than the corrugation obtained considering the global registry index. What is the origin of this difference and what differences in the friction forces calculated by the two methods can this effect lead to?"

Response:

We thank the referee for raising another delicate and important point. The origin of these differences is the fact that the relaxed structures used to calculate the local registry index were obtained via the ILP force field, which was parametrized against non-local many-body dispersion (MBD-NL) corrected DFT calculations with the Heyd-Scuseria-Ernzerhof (HSE) screened hybrid exchange-correlation density functional approximation. Due to the computational burden associated with obtaining HSE+MBD-NL reference date, we opted to parameterize the global registry index against DFT calculations based on the PBE-D3 dispersion corrected exchange-correlation approximation. This leads to small differences in the interlayer distances (i.e., for the AB stacked

MoS_2 bilayer, the HSE+MBD-NL interlayer distance is ~0.2 Å larger than that obtained using PBE-D3), which in turn somewhat affects the resulting sliding energy profile corrugation, without any qualitative effect. A discussion regarding this issue appears above Fig. S1 of the SI. To support the suitability of the PBE-D3 reference data, we added the following sentence in SI section 1: "This approach was shown to capture well the interlayer interactions in TMDs.[4]"

Indeed, the static friction force is proportional to the sliding energy profile corrugation. Hence, using the two reference datasets would result in somewhat different static (and dynamic) friction forces. While both methods are known to provide satisfactory geometries of the systems at hand, it remains to be determined whether HSE+MBD-NL or PBE-D3 provide more accurate sliding potential surfaces. Therefore, extracting frictional information from these two approximations can serve to evaluate the accuracy bounds of the results. Since the present paper focuses on the development of the GRI^{TMD}, regardless of the quality of the reference DFT data, we opt not to go into these details in the revised manuscript. Instead, we note in passing in the caption of Fig. 4 of main text that: "Note that the LRI^{TMD} corrugation of the relaxed structures is somewhat lower than the maximal value obtained when using PBE+D3 DFT based coordinates (see SI section 1). This results from the fact that the ILP was parametrized against non-local many-body dispersion corrected Heyd-Scuseria-Ernzerhof calculations, which produce a somewhat larger interlayer distance (see SI section 4)."

Reviewer 2

"Cao and coworkers report the usefulness of the registry index (RI) to rationalize the frictional properties of layered transition metal dichalcogenides (LTMDs).

The paper opens with a well written, interesting paragraph on the intriguing properties of LTMDs. The authors then go on to investigate if the RI, which is constructed to roughly approximate the repulsion between adjacent layers, correlates with the frictional or transverse forces between the layers. Of course, they do, since resistance to sliding mainly originates from the repulsion between atoms."

Response:

We thank the reviewer for the positive evaluation of our manuscript.

"The positive aspect of the RI is that it is more quantitative than a moiré pattern and more intuitive than a full DFT simulation. This is the point that the authors intend to emphasize. But the real comparison to make is that the RI analysis is less accurate than a full DFT or force-field based simulation and less intuitive than a moiré pattern. And I feel that this is the comparison that needs be made, also because the RI approach needs atomic position as input, so that we need the complexity of a full simulation as input information and then we already know the forces."

Response:

While reading carefully through the remarks of the referee we realized that we might have failed to convey our main message appropriately. Indeed, the RI approach is more quantitative than just viewing a moiré pattern and more intuitive than a full DFT simulation. It is also true that the RI analysis is less accurate than a full DFT or force-field based calculation and less intuitive than the moiré pattern picture. Nonetheless, neither of these messages we set out to convey.

The RI tool should be first and foremost, considered as a quantitative characterization tool. Given an atomistic configuration it allows a simple and extremely intuitive analysis of the global interlayer commensurability, as well as the local interlayer stacking mode patterns. These, in turn, have pronounced manifestations in the physical properties of mismatched layered materials including their frictional, transport, and ferroelectric properties, among others.

Another important aspect of the RI approach is its computational efficiency. Since it is based merely on analytical formulas of circle or Gaussian overlaps, it is extremely efficient and orders of magnitude faster than both force-field and DFT calculations. Notably, this allows one to perform DFT or force-field calculations on small model systems to parametrize an RI expression, and use the latter to perform calculations on large scale systems that are out of the reach of present state-of-the-art dedicated force-fields or DFT approaches. Furthermore, one can also restrict reference sliding data to a one-dimensional path, allowing the fitted RI to extrapolate the higher-dimensional information (see, e.g., Figure 3 of the main text).

It is true also that the RI approach requires input geometries. So do, however, force-field and DFT calculations. Indeed, at present, the RI approach was not used to estimate optimal configurations. This, on its own, is an intriguing endeavor (especially for the case of vertically flexible RI

parameterizations, such as those presented in this paper), which we intend to explore in the future. Having said that, there is a lot of information that can be extracted based on unrelaxed structures using the RI approach. This has been clearly demonstrated when robust superlubricity in heterojunctions of graphene and *h*-BN was first predicted using the RI approach with unrelaxed structures (*J. Phys. Chem. Lett.* **4**, 115-120 (2013)) triggering experimental studies (*Nat. Mater.* **17**, 852-854 (2018)) that verified this prediction and opening a new venue for achieving robust microscale superlubricity, immune to rotational locking into high-friction states. Since then, many computational and experimental groups have followed this idea and demonstrated its generality and applicability in many layered heterojunctions.

As a few concrete examples of the characterization and analysis power of the RI approach one may consider the following three studies: (i) The rationalization of circumferential faceting in multiwalled nanotubes based on interlayer registry considerations (*Nat. Nanotechnol.* **11**, 1082-1086 (2016)); (ii) Explanation of unique vertical transport peaks in twisted graphitic interfaces due to interlayer commensurability variations (*Nat. Nanotechnol.* **11**, 752-757 (2016)); and (iii) Analysis of ferroelectric phenomena in non-centrosymmetric domains of twisted layered interfaces following surface reconstruction (*Science* **372**, 1462-1466 (2021)).

To make these points clearer we have revised the introduction paragraph on page 4 of the revised main text to highlight the predictive, characterization, and analysis power of the registry index approach. The revised paragraph now reads as follows: "Notably, many of these highly complex phenomena can be rationalized in simple geometric terms using the registry index (RI) concept. This approach provides an intuitive and highly computationally efficient geometric measure of the global (GRI) or the spatially resolved local (LRI) interlayer registry of rigid layered material interfaces as they slide atop each other.[15, 28-34] As such, it serves as a compelling characterization tool for commensurability related interfacial phenomena. Previously, the GRI approach was successfully applied to show that the sliding energy landscape of homogeneous and heterogeneous interfaces, such as graphene/graphene, *h*-BN/*h*-BN, MoS_2/MoS_2, graphene/carbon nanotubes, and graphene/*h*-BN, is dictated by the interlayer registry.[15, 28, 30-34] Furthermore, the LRI was shown to be a powerful tool to unveil the physical mechanism underlying circumferential faceting in multiwalled nanotubes,[33] rationalize unique interlayer electronic transport characteristics in twisted graphitic interfaces,[3] and analyze the structural characteristics of reconstructed moiré superlattices in twisted *h*-BN junctions and their relation to the ferroelectric properties of the system.[4]"

"In conclusion, the paper adds neither qualitative insight nor new ways to predict frictional forces without having to run elaborate simulations first. For this reason, I cannot recommend the manuscript for publication, although the presentation itself is excellent."

Response:

We believe that all of the above clearly demonstrates the power of the RI approach and the relevance of its generalization to the case of flexible shifts of parallel and anti-parallel stacked homogeneous and heterogeneous transition metal dichalcogenide interfaces, for the wide readership of the *Journal of Physical Chemistry Letters*.

Reviewer 3

Although Prof. Editor mentioned that we are not obliged to do so, we opted to briefly address the late abridged review.

The paper is technically impeccable and the model seems to provide an excellent interpolation of the DFT results. I am more concerned regarding publication in Journal of Physical Chemistry Letters. Obviously, the idea is not novel, apart from certain technicalities, and I do not see its predictive power for structural properties, let alone for the electronic properties claimed in the introduction. There is only one example presented in the text (the case of a large moiré) and it is not properly described what makes it very difficult to appreciate the real accuracy of the prediction.

Response:

We thank the reviewer for the positive evaluation of the technical aspects of the paper and the validity of our results. The predictive, characterization, and analysis power of the registry index approach have been discussed in detail in our response to Reviewer 2 along the corresponding modifications made in the revised main text. Specifically, the ability of the registry index approach to analyze registry induced ferroelectricity in non-centrosymmetric layered material interfaces is discussed therein.

While the idea behind the registry index approach has been presented by us in the past, the present paper greatly expands its applicability to a large family of homogeneous and heterogeneous interfaces of transition metal dichalcogenides with various stacking configurations allowing the efficient and intuitive evaluation and rationalization of their tribological, transport, and ferroelectric properties (see detailed response to Reviewer 2 above and the corresponding modifications made in the revised manuscript). We believe that this is of high relevance to the wide readership of the *Journal of Physical Chemistry Letters*.

Unfortunately, we fail to understand the last sentence of the abridged report stating that "… it is not properly described what makes it very difficult to appreciate the real accuracy of the prediction.", In fact, we do not recall claiming that it is very difficult to appreciate the real accuracy of our predictions based on the registry index approach.

jz-2021-04202c.R2

Name: Peer Review Information for "The Interlayer Registry Index of Layered Transition Metal Dichalcogenides"

Second Round of Reviewer Comments

Reviewer: 2

Comments to the Author

I thank the authors for their effort to address my criticism. I do realize now better than before that the use of the RI allows one to alleviate the computational cost to predict the resistance to sliding. However, I think that this is somewhat more technical progress than new insight. As referee 1 writes, *… the registry index approach … is extended … to … transition metal dichalcogenides …* as the main distinctive feature of the submitted work compared to previous work. Thus, it could be argued that this work is more of the same. Nonetheless, I would want to give the manuscript a second chance, for a variety of reasons, but also because some of the more technical aspects go beyond previous work and are certainly worth being reported.

To convince me that the RI is useful even without structural relaxation, the authors should provide a discussion, perhaps at a semi-quantitative level, at what length-scale elasticity can no longer be ignored. There is prominent literature discussing this issue, starting with Hirano and Shinjo in their two famous superlubricity papers, followed by a more general scaling analysis proposed by Müser in a 2004 EPL and more recently by Sharp, Pastewka, and Robbins in a 2016 APL simulation study. It seems clear that a two- dimensional adsorbate must eventually pin even when the interface is incommensurate given a sufficiently large size. 2D objects cannot be well approximated as rigid. Quantum fluctuations suffice to destroy the usual scaling of Bragg peaks with system size.

In addition, it might be in place to briefly discuss the ramifications of adsorbates and/or roughness, both of which being essentially unavoidable outside of well-controlled laboratory conditions. Roughness leads to finite contact patches, which were first analyzed quantitatively by Campana in a 2008 Phys Rev E. Unfortunately, his work as well as subsequent work citing his paper deals with semi-infinite elastic objects without intervening flakes and crude estimates of how roughness and the subsequent patchiness of real contact limits the superlubricity of layered compounds. Alternatively, it could be clearly stated that the RI cannot (yet) be applied to rough surfaces.

Last but not least, I have the impression that the concept of the RI was introduced before it was given its name. Kolomogorov and Crespi showed in a 2000 PRL that simple two-body potentials allow commensurability effects in carbon nanotubes to be modeled, Müser, Wenning, and Robbins

demonstrated in a 2001 PRL that a purely repulsive interaction, designed very much in the philosophy of the RI albeit in a continuum rather than in a discrete description, allows the scaling of static friction forces and a function of geometry (disordered or incommensurate, one and two-dimensional, etc) to be predicted. I think that the RI should be introduced or discussed in the context of these and related previous works, which the authors certainly are aware of, assuming they read their own review on superlubricity.

A more minor point: Assuming a good input configuration requires a few dozen or a few hundred force evaluations using DFT or highly-accurate potentials to identify, say, at least the mean separation between the flake and a substrate. How many additional relative configurations were sampled to deduce lateral forces? What I am after is the real speed up due to using the RI. It is probably safe to assume that the RI calculations are cheap compared to an initial relaxation.

In conclusion and as I said before: The writing of the paper is excellent. The technical aspects of the paper are probably superior to previous works. To make me endorse the manuscript, the discussion of the limits of the PI should be improved. I admit that even without this discussion, the paper will attract many citations. However, these will be predominantly from people studying toy models with difficult-to-ascertain relevance to reality and I know that the authors of this manuscript can do better than that.

Author's Response to Peer Review Comments:

Please find attached a cover letter and a detailed response to all comments raised by the referee.

23.03.2022

Dear Prof. Editor,

We thank you for communicating with us the review report on our manuscript (jz-2021-04202c) titled "The Interlayer Registry Index of Layered Transition Metal Dichalcogenides", recently resubmitted for your consideration for publication in the *Journal of Physical Chemistry Letters*.

Attached to this cover letter, please find our point-by-point response to all the comments and suggestions raised by the referee in his/her second report. Also, please find attached the revised manuscript.

We believe that our responses have adequately addressed the referee's comments and we hope that you will share our view that our manuscript, in its revised form, merits publication in the *Journal of Physical Chemistry Letters*.

Sincerely yours,

Oded Hod,

School of Chemistry
Tel-Aviv University
6997801 Tel-Aviv, Israel
Phone: 972-3-640-5850
e-mail: odedhod@tau.ac.il
homepage: http://www.tau.ac.il/~odedhod/

"I thank the authors for their effort to address my criticism. I do realize now better than before that the use of the RI allows one to alleviate the computational cost to predict the resistance to sliding. However, I think that this is somewhat more technical progress than new insight. As referee 1 writes, … the registry index approach … is extended … to … transition metal dichalcogenides … as the main distinctive feature of the submitted work compared to previous work. Thus, it could be argued that this work is more of the same. Nonetheless, I would want to give the manuscript a second chance, for a variety of reasons, but also because some of the more technical aspects go beyond previous work and are certainly worth being reported."

We thank the referee for his positive evaluation of our revised manuscript and for acknowledging that some of the scientific aspects that we report go beyond previous work and hence are worth of being reported.

"To convince me that the RI is useful even without structural relaxation, the authors should provide a discussion, perhaps at a semi-quantitative level, at what length-scale elasticity can no longer be ignored. There is prominent literature discussing this issue, starting with Hirano and Shinjo in their two famous superlubricity papers, followed by a more general scaling analysis proposed by Müser in a 2004 EPL and more recently by Sharp, Pastewka, and Robbins in a 2016 APL simulation study. It seems clear that a two dimensional adsorbate must eventually pin even when the interface is incommensurate given a sufficiently large size. 2D objects cannot be well approximated as rigid. Quantum fluctuations suffice to destroy the usual scaling of Bragg peaks with system size."

We thank the referee for raising this question. In the conclusions section of the revised manuscript we provide a discussion regarding elasticity effects (including appropriate references) and the consequential limitations of the RI approach due to the use of (laterally) rigid model systems. The revised conclusions section now reads as follows: "The ability of the GRI^{TMD} to capture the rigid and vertically relaxed sliding potential energy surfaces of a variety of homogeneous and heterogeneous TMD interfaces indicates the versatility of our approach. A discussion of some possible limitations, however, is in place. Specifically, realistic material interfaces exhibit intra-surface elasticity effects that allow for the adjustment of the slider atoms lateral positions to the underlying potential. Hence, above a critical contact size that depends on the ratio between material elasticity and stiffness of the interfacial interaction, locally commensurate regions may form, resulting in pinning effects and friction enhancement.[44-51] In this respect, a notable advantage of layered materials is their extremely stiff intralayer structure and relatively low inter-layer sliding potential corrugation that may shift the critical length towards larger interface dimensions. Additionally, typical tribological scenarios involve contacts between three-dimensional objects, where interactions with the bulk support may suppress lateral elasticity effects in the contacting surfaces. Other complexities appearing in realistic frictional interfaces include surface roughness and contaminants adsorption, which may also lead to interfacial pinning.[33, 52] The former, may actually break large-scale frictional interfaces into many nanoscale contacts, thus reducing undesirable elasticity effects.[10, 53-54] The latter, can readily be remedied by standard running-in and annealing procedures.[17, 55] Therefore, together with the definition of the LRI^{TMD} that can characterize atomically reconstructed structures[25] the GRI^{TMD} approach provides a simple, intuitive, and

highly computationally efficient approach to treat the structural, tribological, and even ferroelectric,[4] properties of complex TMD interfaces."

"In addition, it might be in place to briefly discuss the ramifications of adsorbates and/or roughness, both of which being essentially unavoidable outside of well-controlled laboratory conditions. Roughness leads to finite contact patches, which were first analyzed quantitatively by Campana in a 2008 Phys Rev E. Unfortunately, his work as well as subsequent work citing his paper deals with semi-infinite elastic objects without intervening flakes and crude estimates of how roughness and the subsequent patchiness of real contact limits the superlubricity of layered compounds. Alternatively, it could be clearly stated that the RI cannot (yet) be applied to rough surfaces."

We thank the referee for providing this comment. We added a discussion regarding possible effects of surface roughness and adsorbates (with appropriate references) in the revised conclusions section of the main text. See revised text in our response to the previous point.

"Last but not least, I have the impression that the concept of the RI was introduced before it was given its name. Kolomogorov and Crespi showed in a 2000 PRL that simple two-body potentials allow commensurability effects in carbon nanotubes to be modeled, Müser, Wenning, and Robbins demonstrated in a 2001 PRL that a purely repulsive interaction, designed very much in the philosophy of the RI albeit in a continuum rather than in a discrete description, allows the scaling of static friction forces and a function of geometry (disordered or incommensurate, one and two-dimensional, etc) to be predicted. I think that the RI should be introduced or discussed in the context of these and related previous works, which the authors certainly are aware of, assuming they read their own review on superlubricity."

Following the referee's suggestion, we have included the relevant references in the introduction section putting the RI approach in context of previous studies. The revised version now reads: Notably, many of these highly complex phenomena can be rationalized in simple geometric terms.[28-37] Specifically, the registry index (RI) approach provides an intuitive and highly computationally efficient geometric measure of the global (GRI) or the spatially resolved local (LRI) interlayer registry of rigid layered material interfaces as they slide atop each other.[15, 35-36, 38-42]

"A more minor point: Assuming a good input configuration requires a few dozen or a few hundred force evaluations using DFT or highly-accurate potentials to identify, say, at least the mean separation between the flake and a substrate. How many additional relative configurations were sampled to deduce lateral forces? What I am after is the real speed up due to using the RI. It is probably safe to assume that the RI calculations are cheap compared to an initial relaxation."

We thank the referee for mentioning this issue. To keep the discussion on a general level, the local nature of atomic centered two-dimensional circle/Gaussian projected overlaps allows the use of

standard neighbor list approaches, which result in $O(N)$ scaling of the RI calculations. This is to be compared with typical N^3 scaling of standard DFT approaches. Therefore, it is indeed safe to assume that the RI calculations are cheap compared to an initial relaxation.

"In conclusion and as I said before: The writing of the paper is excellent. The technical aspects of the paper are probably superior to previous works. To make me endorse the manuscript, the discussion of the limits of the PI should be improved. I admit that even without this discussion, the paper will attract many citations. However, these will be predominantly from people studying toy models with difficult-to-ascertain relevance to reality and I know that the authors of this manuscript can do better than that."

We thank the referee again for his positive evaluation of our revised manuscript and for providing valuable suggestions that allowed us to improve it.
